# Influenza Virus-like Particle-Based Hybrid Vaccine Containing RBD Induces Immunity against Influenza and SARS-CoV-2 Viruses

**DOI:** 10.3390/vaccines10060944

**Published:** 2022-06-14

**Authors:** Ramireddy Bommireddy, Shannon Stone, Noopur Bhatnagar, Pratima Kumari, Luis E. Munoz, Judy Oh, Ki-Hye Kim, Jameson T. L. Berry, Kristen M. Jacobsen, Lahcen Jaafar, Swe-Htet Naing, Allison N. Blackerby, Tori Van der Gaag, Chloe N. Wright, Lilin Lai, Christopher D. Pack, Sampath Ramachandiran, Mehul S. Suthar, Sang-Moo Kang, Mukesh Kumar, Shaker J. C. Reddy, Periasamy Selvaraj

**Affiliations:** 1 Department of Pathology and Laboratory Medicine, Emory University School of Medicine, Atlanta, GA 30322, USA; rbommi2@emory.edu (R.B.); itsluisemg@gmail.com (L.E.M.); jameson.tl.berry@gmail.com (J.T.L.B.); 2 Department of Biology, College of Arts and Sciences, Georgia State University, Atlanta, GA 30303, USA; sstone12@student.gsu.edu (S.S.); pkumari1@student.gsu.edu (P.K.); mkumar8@gsu.edu (M.K.); 3 Institute for Biomedical Sciences, Georgia State University, Atlanta, GA 30303, USA; nbhatnagar1@student.gsu.edu (N.B.); judykjinoh@gmail.com (J.O.); kihyekim4282@gmail.com (K.-H.K.); skang24@gsu.edu (S.-M.K.); 4 Metaclipse Therapeutics Corporation, Atlanta, GA 30340, USA; kjacobsen@metaclipse.com (K.M.J.); jlahcen@metaclipse.com (L.J.); snaing@metaclipse.com (S.-H.N.); ablackerby@metaclipse.com (A.N.B.); tvandergaag@metaclipse.com (T.V.d.G.); cwright@metaclipse.com (C.N.W.); cpack@metaclipse.com (C.D.P.); sramachandiran@metaclipse.com (S.R.); sreddy@metaclipse.com (S.J.C.R.); 5 Department of Pediatrics, Emory Vaccine Center, Yerkes Primate Research Center, Emory University School of Medicine, Atlanta, GA 30322, USA; lilin.lai@emory.edu (L.L.); mehul.s.ssuthar@emory.edu (M.S.S.)

**Keywords:** influenza, virus-like particles, SARS-CoV-2, GM-CSF, RBD, mice, IL-12, antibodies

## Abstract

Several approaches have produced an effective vaccine against severe acute respiratory syndrome coronavirus 2 (SARS-CoV-2). Since millions of people are exposed to influenza virus and SARS-CoV-2, it is of great interest to develop a two-in-one vaccine that will be able to protect against infection of both viruses. We have developed a hybrid vaccine for SARS-CoV-2 and influenza viruses using influenza virus-like particles (VLP) incorporated by protein transfer with glycosylphosphatidylinositol (GPI)-anchored SARS-CoV-2 RBD fused to GM-CSF as an adjuvant. GPI-RBD-GM-CSF fusion protein was expressed in CHO-S cells, purified and incorporated onto influenza VLPs to develop the hybrid vaccine. Our results show that the hybrid vaccine induced a strong antibody response and protected mice from both influenza virus and mouse-adapted SARS-CoV-2 challenges, with vaccinated mice having significantly lower lung viral titers compared to naive mice. These results suggest that a hybrid vaccine strategy is a promising approach for developing multivalent vaccines to prevent influenza A and SARS-CoV-2 infections.

## 1. Introduction

Severe acute respiratory syndrome coronavirus 2 (SARS-CoV-2) first appeared in late 2019 in China before beginning its rapid spread across the globe [1]. The disease, named coronavirus disease of 2019 (COVID-19), presents a severe respiratory disease course and a high fatality rate in the elderly and immunocompromised [2]. The spike (S) protein of the virus binds to the human angiotensin-converting enzyme-2 (ACE2) protein for entry into epithelial cells of the respiratory tract [1,3]. This S protein, specifically the conserved ACE2 receptor-binding domain (RBD), is a proven target for vaccine design. Antibodies and vaccines directed to the S protein and the RBD are effective in preventing SARS-CoV-2 infection [4]. Most of the recent vaccine strategies for SARS-CoV-2 target the full-length S protein (Pfizer/BioNTech, Moderna, Johnson & Johnson, AstraZeneca/Oxford, Novavax, Innovio, Curevac and others) [5,6] and some use inactivated whole virus vaccines (COVAXIN/BBV152 by Bharath Biotech, Sinovac/BBIBP-CorV from Sinopharm) [7,8].

Vaccines for influenza and SARS-CoV-2 have been developed using various platforms and approved by FDA for the general population. Live attenuated viruses, virus-like particles, subunit vaccines, DNA and mRNA vaccines have been tested in the past, but the COVID pandemic created the urgency to develop the mRNA vaccine using lipid nanoparticles for millions of people within a short time. However, the need for affordable vaccines that protect against more than one virus prompted us to develop a two-in-one vaccine since millions of people are exposed to influenza virus in addition to SARS-CoV-2. Preclinical studies evaluating the efficacy of the SARS-CoV-2 vaccine (PiCoVacc, Sinovac Biotech Ltd., Beijing, China) and flu vaccine (Sinovac Biotech Ltd.) in human ACE2 transgenic mice demonstrated that co-administration of two vaccines protected mice from SARS-CoV-2 and influenza virus challenge [9]. Intranasal administration of live attenuated influenza A (LAIV) expressing RBD of SARS-CoV-2 has been shown to prevent SARS-CoV-2 infection in BALB/c mice [10]. However, its efficacy against influenza virus infection was not assessed. Novavax tested a co-administration approach of their SARS-CoV-2 vaccine (NVX-CoV2373) and influenza vaccines in a phase 3 trial and found that most people responded to both vaccines as measured by antibody response and hemagglutination inhibition assay [11]. These studies suggested that it is feasible to develop a vaccine for multiple viruses which can be administered simultaneously.

The present study used a two-in-one hybrid vaccine for SARS-CoV-2 and influenza using a protein transfer method to incorporate cytokine adjuvants and RBD antigen into influenza VLPs. The protein transfer approach achieves a higher level of incorporation of antigen and cytokine adjuvants in the VLP vaccine [12]. Simultaneous the delivery of cytokines and protein antigens by the VLP to antigen-presenting cells (APCs) may enhance the efficient presentation of the antigens to the immune system. Cytokines are known to increase the efficacy of vaccines by attracting and activating key immune cells [13,14,15].

Two cytokines under evaluation for their potential as biological adjuvants are granulocyte-macrophage colony-stimulating factor (GM-CSF) and interleukin-12 (IL-12). In addition, targeting antigens to APCs via a GM-CSF receptor enhances cross-priming [16,17]. GM-CSF potentiates a strong immune response primarily through the maturation and differentiation of dendritic cells [17,18,19,20]. The FDA approved a prostate cancer vaccine, Provenge^®^, by Dendreon, which uses an antigen fused to GM-CSF that delivers antigens effectively to the immune system [21,22]. Adjuvants that have a tolerable safety profile and generate a Th1 immune response are of high importance. Moreover, proinflammatory cytokines produced by activated dendritic cells (DCs) have been shown to play an important role in inducing a robust immune response [23,24]. IL-12 has been well documented to induce a Th1 response, with a promising clinical benefit in cancer patients [13]. IL-12, a heterodimeric cytokine (p35 and p40 subunits), activates DCs, T lymphocytes and natural killer (NK) cells to release IFN-γ, TNF-α, etc. [25,26,27]. IL-12 also induces T-cell precursors to differentiate toward a Th1 lineage, which also promotes the development of a robust CTL response [28]. Preclinical and clinical trials demonstrated the potential of recombinant soluble IL-12 as an adjuvant in treating several cancers and viral hepatitis, resulting in enhanced immune response [14,29,30,31,32,33]; however, it also resulted in unfavorable side effects and systemic toxicity [34,35].

Despite this, delivering IL-12 in a membrane-anchored form has been proved a successful approach in achieving the desired adjuvant effect with minimal toxicity [36,37,38]. We engineered the membrane-bound form of cytokines by attaching a GPI-anchor [39]. The GPI-anchor permits the incorporation of purified GPI-anchored proteins into the lipid bilayer of influenza VLPs or any amphiphilic micro/nanoparticles by a simple protein transfer technique [12,40]. By introducing the membrane incorporated GPI-cytokines into VLPs, viral antigens can be presented to the immune system to mount a robust immune response. In addition, the administration of VLP vaccines containing membrane-anchored cytokines will localize the cytokines to the area of injection, thereby reducing the systemic effects associated with soluble cytokines [12]. The VLP vaccine prepared by our protein transfer approach requires only a low amount of GPI-GM-CSF (25 ng/µg of VLP) for the optimum antiviral response in mice. Moreover, the physical linkage of adjuvant and antigen sources leads to simultaneous adjuvant and antigen delivery to immune cells, resulting in enhanced immune reactivity and increased vaccine efficacy when compared to an unconjugated antigen and adjuvant mixture [41,42]. In the present study, we demonstrate that a hybrid vaccine developed using influenza VLP vaccine incorporated with GPI-RBD-GM-CSF fusion protein and GPI-IL-12 protected mice from influenza virus challenge and also induced a robust, durable antibody response in BALB/c mice as well as decreased viral load and less weight loss when challenged with mouse-adapted SARS-CoV-2.

## 2. Materials and Methods

### 2.1. Antibodies and Proteins

Purified anti-mouse GM-CSF (clone MP1-22E9) and anti-mouse IL-12 (clone C17.8) mAbs were obtained from BioXcell and used for affinity chromatography purification of GPI-RBD-GM-CSF fusion protein and GPI-IL-12, respectively. Anti-RBD mAb (clone MM57) was obtained from Sino Biologicals (Cat#40592). FITC-conjugated goat secondary antibody against mouse IgG/IgM was purchased from BD Pharmingen (Cat# 555988). Peroxidase (HRP)-conjugated goat anti-mouse IgG F(ab’)2 specific antibody was from ThermoFisher Scientific/Pierce (Cat#31436). The antibody isotyping kit was purchased from Southern Biotech (Cat#5300-05). FITC-conjugated streptavidin was purchased from BD Biosciences (Cat#554060). Human COVID-19 convalescent serum samples were purchased from Ray Biotech (Atlanta, GA, USA). HRP-conjugated donkey anti-human IgG antibody was obtained from Jackson Immunoresearch (Cat#709-036-098). Biotinylated human ACE2 from ACRO Biosystems (Cat#AC2-H82F9) and purified RBD protein from Ray Biotech (Cat#230-30162) were purchased.

### 2.2. Mice

BALB/c mice (JAX labs, Bar Harbor, ME or Taconic Biosciences Inc., Germantown, NY, USA) 2–3 months age (female) were purchased and housed in the Emory University Division of Animal Resources (DAR) facility and used according to the University IACUC guidelines.

### 2.3. Construction and Expression of GPI-Anchored SARS-CoV-2 RBD-GM-CSF Fusion Protein

**Figure 1:**
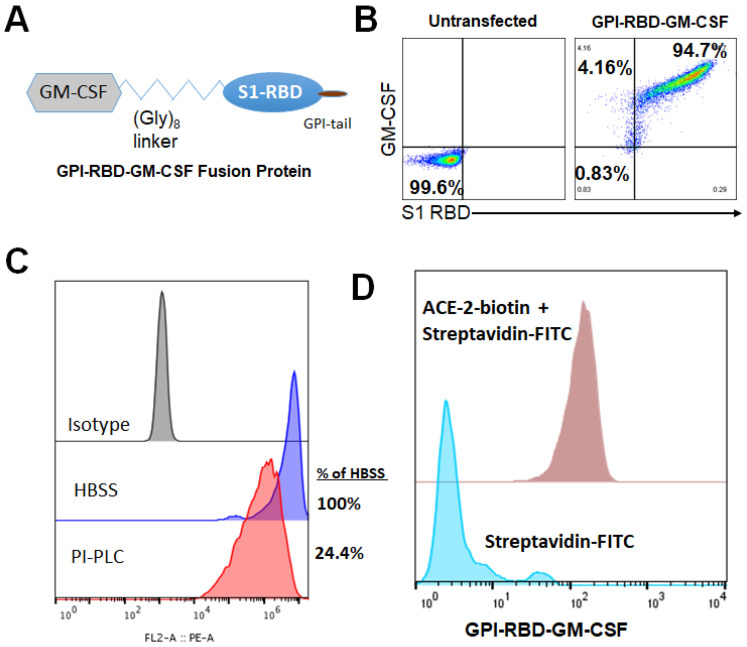
Design, expression and characterization of GPI-RBD-GM-CSF fusion protein. (**A**) Design of GPI-RBD-GM-CSF fusion protein gene, (**B**) GPI-RBD-GM-CSF fusion protein binds both anti-RBD mAb and anti-GM-CSF mAb on CHO-S cell transfectants, (**C**) PIPLC treatment of CHO-S cells expressing GPI-RBD-GM-CSF reduced the level of expression and (**D**) flow cytometry analysis showed binding of human ACE2 to GPI-RBD-GM-CSF fusion protein expressed in CHO-S cells.

We constructed a fusion protein gene by joining the DNA sequences of RBD (amino acids 319–541), mouse GM-CSF and human CD59 GPI-anchor signal sequence as in Figure 1A. This construct was cloned into the pCHO 1.0 vector using the AvrII and BstZ17l sites (Invitrogen). The DNA construct was then transfected into CHO-S cells (Invitrogen) and selected with puromycin and methotrexate. Expression of both the RBD and GM-CSF on the surface of transfected CHO-S cells was confirmed by flow cytometry using fluorophore-conjugated mAbs against RBD, Clone MM57 (Sino Biologicals) and GM-CSF, Clone MP1-22E9 (BioLegend). To confirm whether the fusion protein binds to its cognate receptor ACE2, flow cytometry analysis using biotinylated human ACE2 (ACRO Biosystems) was used and verification that the RBD-GM-CSF fusion protein retains its ability to bind to its cognate receptor was confirmed. CHO-S cell clones transfected with the fusion protein were grown in large quantities using a 5L bioreactor and used for purification by mAb-affinity chromatography.

### 2.4. PIPLC Treatment of CHO-S Cells

To test the ability of phosphatidylinositol-specific phospholipase C (PIPLC) to cleave the GPI-RBD-GM-CSF fusion protein from the CHO-S cells, 0.25 × 10^6^ cells were treated with 0.2 U PIPLC (Sigma cat# 554406) in 250 μL HBSS containing 0.1% BSA for 2 h at 37 °C. After treatment, cells were washed twice with FACS buffer before staining using PE anti-mouse GM-CSF antibody (BD cat# 554406) or PE Rat IgG2a,κ isotype control (BioLegend cat# 400508).

### 2.5. Purification of GPI-RBD-GM-CSF and GPI-IL-12

Frozen CHO-S cell transfectants stably expressing GPI-IL-12 [43] or GPI-RBD-GM-CSF fusion protein were lysed for 1 h at 4 °C in lysis buffer (50 mM Tris, 20 mM Iodoacetamide, 5 mM EDTA, 0.2% Tween 20, 2 mM PMSF and protease inhibitor cocktail, pH 8.0) and membranes containing the GPI-proteins were collected by centrifugation at 17,000× *g* for 1 h at 4 °C. The membranes were lysed with octyl glucoside [40], and GPI-RBD-GM-CSF and GPI-IL-12 present in the lysates were purified using anti-mouse GM-CSF antibody (Clone MP1-22E9, BioXCell) and anti-mouse IL-12 antibody (clone C17.8) coupled to NHS-Sepharose affinity (Cytiva) columns, respectively.

### 2.6. SDS-PAGE and Western Blot

Proteins were separated on a NuPAGE^TM^ 12% Bis-Tris Gel, 1.0 mm × 10 well (Cat# NP0341BOX), and stained with Invitrogen’s Colloidal Blue Staining Kit, “Stain NuPAGE, Novex Bis-Tris Gel” (catalog # 46-7015, 46-7016), following manufacturer’s instructions. For Western blotting, proteins separated by 12% sodium dodecyl sulphate-polyacrylamide gel electrophoresis (SDS-PAGE) under non-reducing condition were transferred onto a nitrocellulose membrane using semi-dry transfer apparatus via an electrical current. After transfer, the membranes with the proteins were blocked for 1 h at room temperature with 5% milk in phosphate-buffered saline and 0.2% Tween 20 (PBS-T) and incubated with a primary antibody (anti-RBD, anti-mouse GM-CSF or anti-mouse IL-12) overnight at 4 °C in PBS-T with on a shaker at low speed. The next day, membranes were washed three times with PBS-T and then incubated with an appropriate secondary antibody conjugated with alkaline phosphatase that provides a visual color change upon the addition of the chromogenic substrate (mixture of BCIP (5-bromo-4chloro-3-indolyl phosphate- catalog# 34040) and NBT (nitro-blue tetrazolium chloride, catalog# 34035 from Thermo Scientific)).

### 2.7. Hybrid Vaccine Preparation by Protein Transfer of GPI-RBD-GM-CSF Fusion Protein and GPI-IL-12 onto VLP

Influenza VLPs containing codon-optimized hemagglutinin (H1 HA) and matrix M1 proteins derived from A/Puerto Rico/8/1934 (PR8) were produced in insect cells (Sf9) as described in [44] and purified by tangential flow diafiltration and anion exchange (Capto Q) chromatography by Medigen (Frederick, MD, USA). We incorporated the purified GPI-RBD-GM-CSF fusion protein along with GPI-IL-12 into PR8 influenza VLPs by protein transfer to prepare our VLP-RBD-GM-CSF-IL-12 vaccine. Protein transfer was performed by incubating 1 mg of enveloped influenza VLPs with 200 μg of purified GPI-RBD-GM-CSF and 25 μg of purified GPI-IL-12 at 37 °C for 1 h. Unincorporated GPI-cytokines were washed out by ultracentrifugation at 210,000× *g* at 4 °C, and the resulting pellet was resuspended in DPBS. Protein incorporation was detected by Western blot and flow cytometry analysis using anti-RBD mAb, clone MM57 (Sino Biologicals), anti-mouse GM-CSF (clone MP1-22E9, BioLegend, San Diego, CA, USA) and anti-mouse IL-12 (clone C17.8, Invitrogen, Rochester, NY, USA) antibodies.

### 2.8. Bone Marrow-Derived Dendritic Cell Stimulation Assay

BMDCs were generated according to established protocols [45]. Briefly, femurs of female BALB/c mice were removed and cleaned from surrounding muscle tissue. Bone marrow was flushed using RPMI-1640 medium with a 22 G needle and syringe. Red blood cells (RBC) were lysed using RBC lysis buffer (MilliporeSigma, Burlington, MA, USA), and the resulting cells were cultured in a complete RPMI-1640 medium containing various concentrations of recombinant murine GM-CSF (rGM-CSF, BioLegend, San Diego, CA, USA), GPI-GM-CSF, GPI-RBD-GM-CSF or VLPs incorporated with GPI-RBD-GM-CSF at a density of 2 × 10^5^ cells/mL. After 3 days of culture, 2,3-bis-(2-methoxy-4-nitro-5-sulfophenyl)-2H-tetrazolium-5-carboxanilide (XTT) assay reagent was added, and absorbance was measured after 3 h at 480 nm and 660 nm for background reference.

### 2.9. Immunization with VLP Vaccine

BALB/c mice were administered with VLP vaccine, control VLP or PBS subcutaneously (100 μL volume per mouse) or intramuscularly (50 μL per mouse in the hind leg). A booster dose was administered 2 or 4 weeks. VLP vaccines were diluted in sterile PBS before administering to the mice.

### 2.10. Enzyme-Linked Immunosorbent Assay (ELISA)

SARS-CoV-2 S protein RBD specific antibodies of different subtypes (IgG, IgG1, IgG2a) were determined in sera by enzyme-linked immunosorbent assay (ELISA) using an approach similar to one previously described using PR8 or WSN as targets [46,47]. Briefly, 96-well ELISA plates were coated with 100 μL of 3 μg/mL GPI-RBD-GM-CSF in coating buffer (BioLegend) overnight at 4 °C. In some experiments, RBD of spike protein from commercial sources (RayBiotech, Atlanta, GA, USA or Sino Biologicals, Wayne, PA, USA) was used as indicated in the figure legends. Plates were washed using a plate washer and washing buffer (PBS with 0.05% Tween20). Plates were blocked with assay diluent (PBS with 3% BSA) for 2 h at room temperature on a rocker. Plates were washed again as described above, and diluted serum samples were added and incubated for another 2 h at room temperature. Plates were washed, and diluted secondary antibody (HRP-conjugated) against mouse total IgG or immunoglobulin isotypes (IgG1, IgG2a) was added and incubated for 30 min at room temperature. The plates were washed, and TMB substrate solution (Cat# 555214, BD Biosciences) was added to the wells for color development. The reaction was stopped by adding 2N H_2_SO_4_ and reading the absorbance at 450 nm.

To measure influenza antigen-specific antibody levels in immune sera, inactivated A/PR8 H_1_N_1_ virus (200 ng/well) was coated onto ELISA plates, followed by the addition of diluted immune sera. IgG isotypes were measured using goat anti-mouse immunoglobulin (Ig) G, IgG1 and IgG2a and horse-radish peroxidase (HRP)-conjugated secondary antibodies (Southern Biotechnology, Birmingham, AL, USA). Color reactions were developed with tetramethylbenzidine substrates (TMB, Invitrogen). Antibody levels are presented as optical density absorbance values at 450 nm (BioTek ELISA plate reader, Winusky, VT, USA).

### 2.11. Flow Cytometry Analysis of Antibody Response

For the detection of RBD-GM-CSF fusion protein on CHO-S cells, CHO-S cells (2–3 × 10^6^/mL) were incubated with anti-mouse GM-CSF antibody, anti-RBD (clone MM57, Sino Biological) in FACS buffer (PBS containing 2% BCS, 5 mM EDTA and 0.05% sodium azide), for 30–60 min on ice. For mouse sera, CHO-S cells were incubated with diluted serum samples (100–100,000 times diluted in FACS buffer). FITC-conjugated goat anti-mouse IgG/IgM was added as a secondary antibody after washing off the unbound primary antibody. After incubation with secondary antibody, cells were washed with FACS buffer and resuspended in FACS buffer and acquired in a FACSCalibur (BD Biosciences) flow cytometer. Data were analyzed using FlowJo software (BD Biosciences, Ashland, OR, USA).

### 2.12. Focus Reduction Neutralization Assay

Live-virus SARS-CoV-2 neutralization antibodies were assessed using a full-length mNeonGreen SARS-CoV-2 (2019-nCoV/USA_WA1/2020), generated as previously described [48]. FRNT-mNG assays were performed as previously described [49]. Briefly, samples were diluted at 3-fold in 8 serial dilutions using DMEM (VWR, #45000-304) in duplicates with an initial dilution of 1:10 in a total volume of 60 μL. Serially diluted samples were incubated with an equal volume of SARS-CoV-2-mNG (100–200 foci per well) at 37 °C for 1 h in a round-bottomed 96-well culture plate. The antibody-virus mixture was then added to Vero cells and incubated at 37 °C for 1 h. Post-incubation, the antibody-virus mixture was removed, and 100 µL of prewarmed 0.85% methylcellulose (Sigma-Aldrich, #M0512-250G) overlay were added to each well. Plates were incubated at 37 °C for 24 h. After 24 h, the methylcellulose overlay was removed, and cells were washed three times with PBS. Cells were then fixed with 2% paraformaldehyde in PBS (Electron Microscopy Sciences) for 30 min. Following fixation, plates were washed twice with PBS and foci were visualized on a fluorescence ELISpot reader (CTL ImmunoSpot S6 Universal Analyzer) and counted using Viridot [50]. The neutralization titers were calculated as follows: 1-(ratio of the mean number of foci in the presence of sera and foci at the highest dilution of the respective sera sample). Each specimen was tested in duplicate. The FRNT-mNG50 titers were interpolated using 4-parameter nonlinear regression in GraphPad Prism 8.4.3.

### 2.13. Plaque Reduction Neutralization Test (PRNT)

The titers of anti-SARS-CoV-2 neutralizing antibodies were measured in the serum of BALB/c mice using a PRNT assay as described previously [51]. Serum was diluted serially from 1:4 to 1:1024, and PRNT was conducted using the Wuhan strain of SARS-CoV-2 (BEI NR-52281). The highest dilution of serum resulting in a 50% reduction in the number of plaques compared to the growth of the virus control was determined.

### 2.14. Hemagglutination Inhibition (HAI) Assay

To assess the ability of immune sera to inhibit HA activity, we performed an HAI assay using immune sera. The immune sera from each group were treated with receptor destroying enzymes (RDE, Sigma-Aldrich, St. Louis, MO, USA) for 18 h at 37 °C. Sera were incubated at 56 °C for 30 min for the inactivation of Complement, followed by 10-fold serial dilutions in PBS. The serially diluted sera were incubated with 4 HA units of the A/PR8 H1N1 virus for 30 min at room temperature, and then 0.5% chicken red blood cells (Lampire Biological Laboratories) were added to determine HAI titers. HAI titers were determined as the highest dilution factor inhibiting the formation of buttons with 0.5% chicken red blood cells.

### 2.15. SARS-CoV-2 Virus Challenge

Female BALB/c mice (10-week-old) were purchased from Taconic Biosciences and housed in the Emory University DAR facility. Mice were immunized with the VLP or VLP vaccine with cytokine adjuvants containing RBD and administered a booster dose 33 days after the first dose (*n* = 10 mice per treatment). Blood was collected 2 weeks after the booster dose for the antibody titer. Mice were transferred 3 months after the booster dose to ABSL-2 (*n* = 5 for each treatment) for challenge with influenza A virus or ABSL-3 (*n* = 5 for each treatment) for challenge with mouse-adapted SARS-CoV-2 virus MA10 at the Georgia State University (GSU). All the animal infection experiments using the SARS-CoV-2 virus were conducted in a certified Animal Biosafety Level 3 (ABSL-3) laboratory at GSU. The protocol was approved by the GSU Institutional Animal Care and Use Committee (Protocol number A20044). MA10 virus is not 100% lethal in young BALB/c mice. Around 50% of animals clear the virus and survive the infection. Peak virus titers in the lungs are detected between days 2 and 4 after the infection. No virus is detected in the animals that survive the infection. Therefore, we euthanized all the animals on day 3 to compare the viral load in the lungs. Mice were inoculated intranasally with 10^5^ plaque-forming units (PFU) of mouse-adapted SARS-CoV-2 MA10 [52,53]. Animals were weighed, and their appetite, activity, breathing and neurological signs were assessed twice daily. On day 3 after infection, animals were anesthetized with isoflurane and perfused with cold PBS. Lungs were collected and flash-frozen in 2-methylbutane (Sigma, St. Louis, MO, USA).

### 2.16. Quantification of the SARS-CoV-2 Virus Load in the Lungs

Quantitative RT-PCR was used to measure viral RNA levels with primers and probes specific for the SARS-CoV-2 N gene as described previously [51]. Viral genome copies were determined by comparison to a standard curve generated using a known amount of RNA extracted from previously titrated SARS-CoV-2 samples. Frozen tissues harvested from mock and infected animals were weighed and lysed in RLT buffer (Qiagen), and RNA was extracted using a Qiagen RNeasy Mini kit (Qiagen, Germantown, MD, USA). Total RNA extracted from the tissues was quantified and normalized, and viral RNA levels per μg of total RNA were calculated.

### 2.17. Influenza A/PR8 H_1_N_1_ Virus Challenge

Three months after the booster dose, BALB/c mice were challenged with a lethal dose of influenza A/PR8 H_1_N_1_ virus (10× LD_50_). After challenge, the mice were monitored for 14 days to record body weight changes and survival rates. To determine the protective efficacy and T cell responses after A/PR8 H1N1 infection, an additional set of immunized BALB/c mice was euthanized on day 5 post-infection, and lung tissues were collected for further analysis.

### 2.18. Influenza Virus Titration in the Lung

The lungs of the immunized BALB/c mice were harvested on day 5 after A/PR8 H1N1 infection and ground mechanically in 1.5 mL of PBS per lung. The lung extracts and lung cells were separated after centrifugation. Embryonated chicken eggs (Hy-Line North America, LLC, Wilton, IA, USA) were incubated at 37 °C for 9–12 days to be inoculated with 10-fold serially diluted lung extracts. The virus titers were determined by hemagglutination assay of the allantoic fluids collected after 3 days of incubation. Virus titers as 50% egg infection dose (EID_50_)/mL were evaluated according to the Reed and Muench method [54].

### 2.19. Statistical Analysis

GraphPad Prism (version 9.1.0) was used to generate the graphs and analysis of the statistical significance as indicated in the figure legends. A *p*-value < 0.05 was considered significant.

## 3. Results

### 3.1. Characterization of GPI-RBD-GM-CSF Fusion Protein

We constructed a fusion protein gene by joining the DNA sequences specific to the SARS-CoV-2 S protein RBD domain, mouse GM-CSF and the GPI-anchor signal from human CD59 (Figure 1A) and expressed the gene in CHO-S cells. Flow cytometry analysis demonstrated the expression of the RBD-GM-CSF fusion protein on the surface of transfected CHO-S cells (Figure 1B). To verify whether the GPI-RBD-GM-CSF fusion protein contained the GPI tail, CHO-S cells expressing the fusion protein were incubated with phosphatidylinositol-specific phospholipase C (PIPLC), which is known to cleave the GPI moiety from the proteins and release them from the cell membranes as described in Methods. The flow cytometry data show that nearly 76% of the fusion protein was released from the cell surface, suggesting that the fusion protein is anchored to the cell membrane via the GPI tail (Figure 1C). To determine whether the fusion protein on the CHO-S cell surface binds to its cognate receptor ACE2, flow cytometry analysis was used to detect the binding of biotinylated human ACE2 (ACRO Biosystems) to the CHO-S cells. The results verified that the RBD in the fusion protein retained its ACE2 binding activity (Figure 1D). RBD-specific neutralizing antibody (clone MM57, Sino Biologicals) was able to bind to GPI-RBD-GM-CSF on CHO-S cell transfectants, which was blocked by pre-incubation with purified GPI-RBD-GM-CSF and commercially available RBD (RayBiotech), confirming that the fusion protein retains the RBD conformation (Figure S1). Transfected cells were cloned, and CHO-S clone 3C3 was used for further studies.

### 3.2. GPI-RBD-GM-CSF Fusion Protein Retains Functional Activity

**Figure 2:**
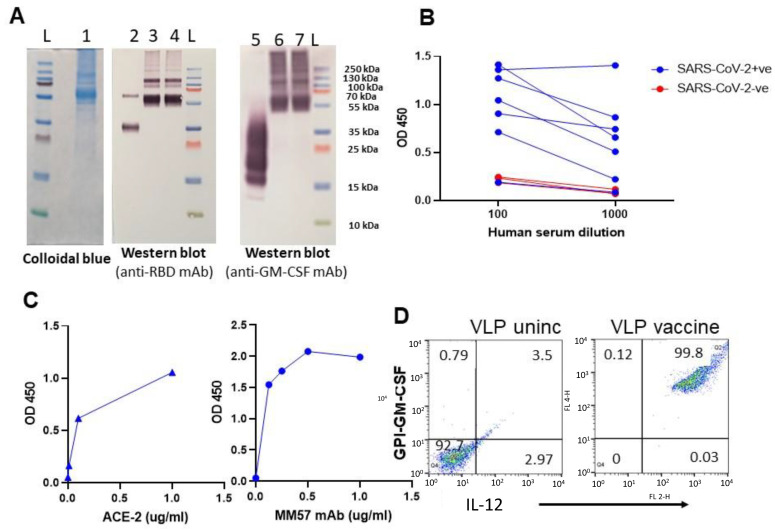
Purified GPI-RBD-GM-CSF fusion protein retains functional activity. (**A**) Colloidal blue (lane 1) and Western blot (lanes 2–7) of the immunoaffinity column purified fusion protein from CHO-S cells probed with anti-RBD antibody (lanes 3 and 4) or anti-GM-CSF mAb (lanes 6 and 7). Lane 2 is control RBD probed with anti-RBD Ab, and lane 5 is control GM-CSF probed with anti-GM-CSF antibody. (**B**) ELISA for GPI-RBD-GM-CSF binding to antibodies in the human COVID-19 patients’ sera, and (**C**) ELISA of purified GPI-RBD-GM-CSF binding to ACE2 and RBD specific MM57 mAb. (**D**) FACS analysis of VLPs incorporated with GPI-IL-12 and GPI-RBD-GM-CSF fusion protein by protein transfer.

CHO-S cell-expressed GPI-RBD-GM-CSF fusion protein was affinity purified using NHS-Sepharose coupled to an anti-mouse GM-CSF antibody. The purified fusion protein was run on 12% SDS-PAGE under non-reducing conditions and either stained with Colloidal blue (Figure 2A, lane 1) or detected with Western blots using an anti-RBD antibody (Figure 2A, lanes 3 and 4) and anti-GM-CSF (Figure 2A, lanes 6 and 7). Affinity purified GPI-RBD-GM-CSF fusion protein runs as a smear ranging from 50 kDa (size of non-glycosylated fusion protein) to 250 kDa with several distinct bands with sizes 55 kDa, 110 kDa and 220 kDa. Western blot analysis was performed for identity and size comparison of the fusion protein to wild-type mouse GPI-GM-CSF (Figure 2A, lane 5). Most of the colloidal blue-stained bands were detected by anti-mouse GM-CSF and anti-RBD antibodies, suggesting that they are multimeric forms of the fusion protein.

To test whether the anti-RBD antibodies in the convalescent sera from SARS-CoV-2 infected patients (RayBiotech) recognize the RBD in the purified GPI-RBD-GM-CSF fusion protein, we performed a direct ELISA. The data show that antibodies from COVID-19 patients’ sera bind to the GPI-RBD-GM-CSF (Figure 2B). Next, we tested whether the purified fusion protein has a dual function as GM-CSF and also binds human ACE2. To test whether GM-CSF in the fusion protein retains its function, we cultured mouse bone marrow-derived dendritic cells (BMDC) in vitro with purified GPI-RBD-GM-CSF and measured BMDC proliferation using an XTT assay. The results show that GPI-RBD-GM-CSF is capable of stimulating BMDC proliferation (Figure S2). An ELISA using biotinylated human ACE2 confirmed that the RBD in purified GPI-RBD-GM-CSF fusion protein retains its ACE2 receptor binding activity (Figure 2C left panel). RBD-specific neutralizing antibody MM57 was used as a positive control (Figure 2C right panel).

To develop the VLP hybrid vaccine, we incorporated influenza VLPs with GPI-IL-12 and GPI-RBD-GM-CSF by protein transfer. Influenza VLPs contain a lipid bilayer which is amenable to protein transfer mediated incorporation of GPI-proteins. Flow cytometry was performed using fluorochrome-conjugated anti-IL-12 and anti-GM-CSF antibodies to confirm the dual incorporation of the GPI-RBD-GM-CSF and GPI-IL-12 onto VLPs (Figure 2D). To test whether VLP-incorporated GM-CSF retains its function, we cultured mouse bone marrow-derived dendritic cells (BMDC) in vitro with soluble GPI-RBD-GM-CSF or VLPs incorporated with GPI-RBD-GM-CSF and measured BMDC proliferation using an XTT assay. The results show that GPI-RBD-GM-CSF incorporated in the VLP vaccine is capable of stimulating BMDC proliferation (Figure S2).

### 3.3. Hybrid Vaccine Induces Durable Antibody Response

To test whether the GPI-RBD-GM-CSF fusion protein induces antibody response, we immunized BALB/c (2–3 months old) mice with GPI-RBD-GM-CSF fusion protein (0.1, 1.0, 2.0 and 5.0 μg) or VLPs (1.0, 2.0, 5.0 and 10 μg) incorporated with the fusion protein and GPI-IL-12 (hybrid vaccine). Controls included VLP without cytokines, commercially available RBD (RBD-His from Ray Biotech) or PBS. A booster dose was administered 2–4 weeks after the first dose. The route of administration was either subcutaneous (s.c.) or intramuscular (i.m.) (Figure S3).

Blood was collected every 2 to 4 weeks for antibody titer, ACE2 binding inhibition and virus neutralization assays. The VLP hybrid vaccine induced a robust antibody response after the booster dose (Figure S4). The antibody response against GPI-RBD-GM-CSF fusion protein was comparable in both i.m. and s.c. routes of vaccine administration (Figure S5). The antibodies bind to CHO-S cells expressing the GPI-RBD-GM-CSF fusion protein but not untransfected CHO-S cells (Figure S6), confirming the specificity of the antibody response to GPI-RBD-GM-CSF fusion protein. 

### 3.4. Hybrid Vaccine Induces SARS-CoV-2 Neutralizing Antibodies

To test whether antibodies generated by the hybrid vaccine block the binding of RBD to human ACE2, we performed an inhibition assay using biotinylated ACE2 and CHO-S cells expressing GPI-RBD-GM-CSF fusion protein. Our results show that antibodies induced by both purified fusion protein and hybrid vaccine blocked ACE2 binding to RBD on CHO-S cells (Figure S7). We used ACE2 blocking anti-RBD (clone MM57) mAb (2μg and 10 μg) as a positive control. For live virus neutralization, Vero.E6 cells and the WA1 strain of SARS-CoV-2 were used in a modified FRNT assay as described in a previously published study [49]. While the purified GPI-RBD-GM-CSF induced levels of antibody response similar to that of the VLP hybrid vaccine (Figure S8A), neutralizing antibody titers were very low in the mice that received only the purified fusion protein GPI-RBD-GM-CSF without VLP. The data suggest that VLP incorporated fusion protein induced stronger neutralizing antibodies than the soluble fusion protein (Figure S8B).

### 3.5. Hybrid Vaccine Induces IgG2a Antibody Response against RBD

**Figure 3:**
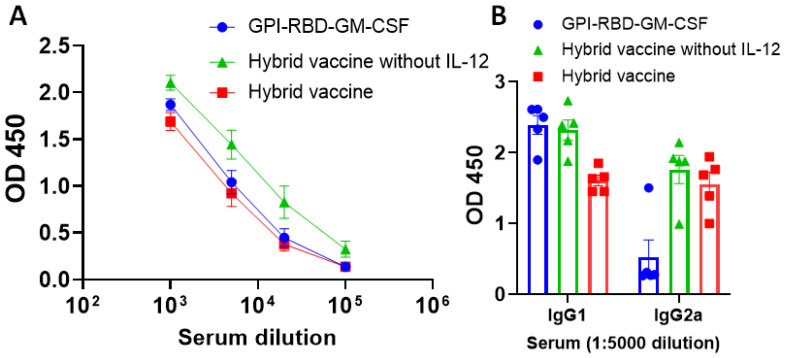
Hybrid vaccine induces antibody response against RBD in mice. ELISA plates were coated with GPI-RBD-GM-CSF, and serum samples from various groups of mice (*n* = 5) immunized with GPI-RBD-GM-CSF, VLP vaccine containing the GPI-RBD-GM-CSF or hybrid vaccine (VLP vaccine containing the GPI-RBD-GM-CSF + GPI-IL-12) were diluted and added to the wells after blocking the plates. (**A**) Total IgG levels 6 weeks after booster dose, and (**B**) IgG isotype in the sera 10 weeks after the booster dose. Anti-mouse IgG (**A**) or isotype-specific anti-mouse IgG-HRP conjugate (**B**) was used to detect the bound antibody.

We observed that purified GPI-RBD-GM-CSF fusion protein by itself or incorporated onto VLPs induced a strong antibody response (Figure 3A). However, the recombinant RBD-His tag failed to induce an antibody response, suggesting that the GM-CSF in our fusion protein is acting as an adjuvant (Figure S9). To determine the isotype of antibodies induced by the hybrid vaccine, we performed an antibody isotyping ELISA as described in the Section 2. While the purified GPI-RBD-GM-CSF fusion protein alone induced an antibody response, which is mostly Th2 type IgG1 (blue circles, Figure 3B), the hybrid VLP vaccine induced both IgG2a (a Th1-induced response) and IgG1 (red symbols, Figure 3B). However, the addition of GPI-IL-12 to the VLP vaccine did not further enhance the IgG2a response (green versus red bars).

### 3.6. Hybrid Vaccine Induces Anti-Influenza Virus IgG1 and IgG2a Antibody Response

**Figure 4:**
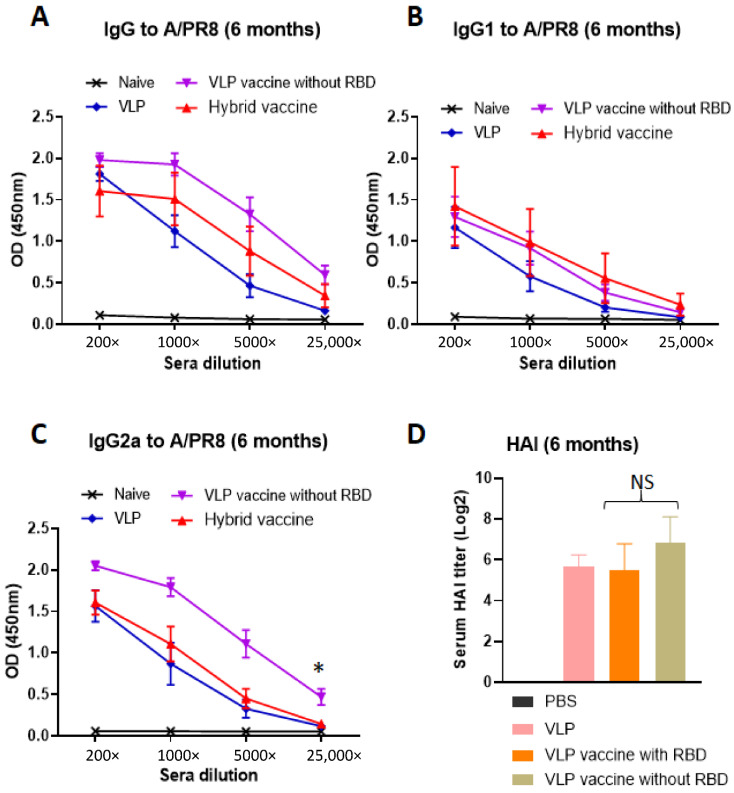
Hybrid vaccine induces influenza A/PR8 virus-specific antibody response. (**A**–**C**) ELISA plates were coated with inactivated influenza A/PR8 H1N1. Serum samples from mice vaccinated with VLP, VLP with GPI-GM-CSF and GPI-IL-12 or hybrid vaccine were serially diluted (5-fold) and added to the wells after blocking the plates. (**D**) Hemagglutination inhibition (HAI) titer in the sera of mice. Isotype specific anti-mouse Ig-HRP conjugate was used to detect the isotype of the antibody bound. * *p* < 0.05.

To test whether the hybrid vaccine-induced antibodies against influenza virus antigens, we analyzed the sera for antibodies and the isotype of the antibodies. The VLP vaccine incorporating the cytokines (purple and red symbols) induced higher levels of antibodies compared to the VLP without cytokines (blue symbols, Figure 4A). The antibody response is a mixed Th1 and Th2 type (IgG1 and IgG2a) against influenza A/PR8 antigens in inactivated virus (Figure 4A–C). Interestingly, we observed that the VLP vaccine without RBD-GM-CSF that was incorporated with GPI-GM-CSF instead (purple symbols) induced significantly higher levels of IgG2a (Figure 4C) but not IgG1 (Figure 4B) compared to the VLP administered group suggesting that GPI-IL-12 and GPI-GM-CSF in the VLP vaccine augment a Th1-type IgG2a antibody response. The incorporation of GPI-RBD-GM-CSF instead of GPI-GM-CSF onto the VLP vaccine diminished the level of total anti-influenza antibody response (red symbols in Figure 4C). This may be because GPI-GM-CSF is more active than GPI-RBD-GM-CSF or may be due to the difference in the level of incorporation onto VLP or both, which requires further investigation. However, the hemagglutination inhibition (HAI) antibody titer induced by the VLP vaccine with RBD and VLP vaccine without RBD vaccines are comparable (Figure 4D), suggesting the differences in IgG2a antibody are probably due to non-neutralizing antibodies induced against influenza A/PR8 VLPs. Our results also show that the VLP vaccine with GPI-RBD-GM-CSF with and without GPI-IL-12 induced equally potent antibody responses against inactivated influenza A/PR8 virus (Figure S10).

### 3.7. Hybrid Vaccine Protects Mice from SARS-CoV-2 and Influenza Virus Challenges

**Figure 5:**
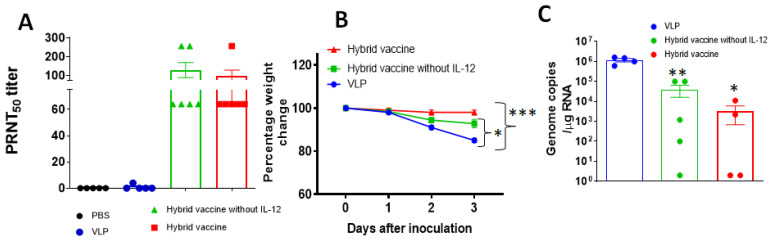
Hybrid vaccine protects against SARS-CoV-2 challenge. BALB/c mice (*n* = 10; 2–3 months old) were administered with a hybrid vaccine, a hybrid vaccine without IL-12 or VLP in 50 ul volume via an intramuscular route. Control mice received either PBS or influenza VLP. A booster dose was given on d33 and blood was collected 2 weeks later (week 7). Mice were challenged with mouse-adapted SARS-CoV2 (*n* = 5) 16–18 weeks after the first dose. (**A**) Neutralizing antibody titers in the serum of BALB/c mice (*n* = 5–6 per group). Serum collected from BALB/c mice 2 weeks after the booster dose was serially diluted from 1:4 to 1:1024, and PRNT was conducted against SARS-CoV-2 (Wuhan virus). (**B**) BALB/c mice were inoculated intranasally with mouse-adapted SARS-CoV-2 (10^5^ plaque-forming units) 3 months after the booster dose. Percentage of daily body weight change in the animals. (**C**) The RNA levels of SARS-CoV-2 were determined in the lungs by qRT-PCR (*n* = 4–5 per group). Error bars represent SEM. The data are expressed as genome copies/μg of RNA. Each data point represents an individual mouse. Data are expressed as mean log_10_ titer. For body weight changes, a two-way analysis of variance (ANOVA) with the post hoc Bonferroni test was used to calculate values of p. Mann–Whitney test was used to calculate the *p* values of the difference between viral titers. Differences of *p* < 0.05 were considered significant. * *p* < 0.05; ** *p* < 0.01 *** *p* < 0.001. Hybrid vaccine: VLP incorporated with GPI-RBD-GM-CSF and GPI-IL-12.

To test whether the vaccine administration induces protective response, mice were immunized with unmodified VLP, hybrid vaccine without IL-12 and hybrid vaccine. A booster dose was administered after 33 days after the first dose. Blood was collected 2 weeks after the booster dose, and anti-RBD antibody response against RBD and influenza virus antigens and neutralizing antibody titers using live SARS-CoV-2 (Wuhan strain) were performed as described in the Section 2. Our results indicate that the hybrid vaccine and hybrid vaccine without IL-12 induced strong antibody responses against RBD (Figure S11A) and influenza VLP antigens (Figure S11B) but did not bind to recombinant GM-CSF (Figure S11C). The antibodies were able to neutralize live virus infection in a plaque reduction neutralization titer (PRNT) assay (Figure 5A). These mice were transferred to the ABSL3 facility for challenging with mouse-adapted SARS-CoV-2 virus (MA10). Mice were challenged with 10^5^ plaque-forming units of the SARS-CoV-2 virus MA10. We observed acute weight loss in control VLP administered mice, but the mice that were administered with either the hybrid vaccine or hybrid vaccine without IL-12 were protected from weight loss (Figure 5B). Since this virus is not lethal for mice, mice were euthanized for quantification of lung viral titer after 3 days of challenge. Virus titer estimates revealed that the hybrid vaccine decreased virus replication significantly compared to the mice that received VLP. The hybrid vaccine that contained IL-12 in addition to RBD-GM-CSF fusion protein was more effective in controlling lung viral loads than the hybrid vaccine without IL-12 (Figure 5C).

**Figure 6:**
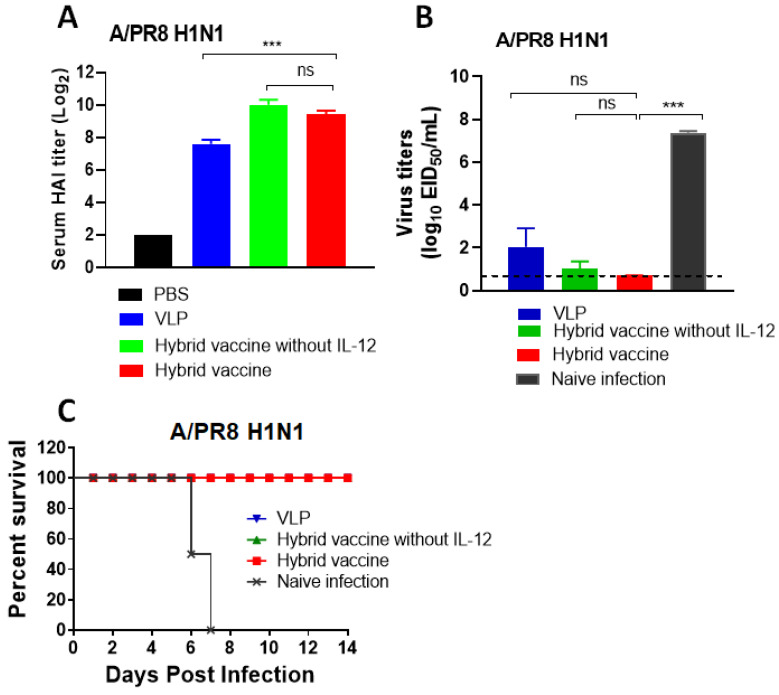
Hybrid vaccine protects against influenza A virus challenge. BALB/c mice were administered with a hybrid vaccine (10 μg/dose) or a hybrid vaccine without GPI-IL-12, as described in Figure 5. A booster dose was given after 33 days of the first dose. (**A**) HAI titer in the blood 2 weeks after the booster dose (day 48). Lung viral titer (**B**) and survival (**C**) of mice challenged with influenza A/PR8 H1N1 virus 3 months after the booster dose. The inoculation dose was 10 times LD_50_. (C) All three groups (VLP, hybrid vaccine and hybrid vaccine without IL-12) survived from PR8 challenge; therefore, the lines are superimposed, and only the red symbol is visible. Hybrid vaccine: VLP incorporated with GPI-RBD-GM-CSF and GPI-IL-12. Statistical significance was calculated by one-way ANOVA and Dunnett’s post-multiple comparison tests. Error bars indicate the mean ± standard errors of the mean (SEM). ***; *p* < 0.001, ns; not significant.

Another cohort of mice from the same treatment groups was transferred to the ABSL2 facility for influenza A/PR8 virus challenge. Hemagglutination inhibition titers against the A/PR8 virus were determined before challenge. The results showed that VLP and hybrid vaccine administration induced high titers of hemagglutination inhibiting antibodies (Figure 6A). Mice were challenged with 10 LD_50_ of influenza A/PR8 and monitored for their body weight changes and survival rates. Since the mice administered with VLP or VLP vaccines were protected from weight loss compared to control infected mice (data not shown), we euthanized three mice from each group after 5 days of infection and measured A/PR8 H1N1 titers in the lungs. We observed that the viral loads were significantly reduced up to a level of detection limit in mice that received VLP, hybrid vaccine or hybrid vaccine without IL-12 (Figure 6B) compared to the PBS control. The virus was undetectable in mice administered with a hybrid vaccine, but VLP alone was also equally protective against influenza virus. Consistent with the viral titers, the mice vaccinated with VLP or VLP with cytokine adjuvants survived the lethal challenge (Figure 6C).

## 4. Discussion

Developing vaccines using nanomaterials such as lipid nanoparticles (LNPs) to increase the efficacy and stability of mRNA vaccines was a breakthrough in recent times. LNPs not only enhance the stability of the viral mRNA but also act as adjuvants [55,56]. Virus-like particles (VLPs) are also considered nanomaterials because of their size (10–200 nm) [57]. These are successfully used to deliver multiple antigens as vaccines [58,59]. Influenza VLPs are lipid enveloped nanoparticles and therefore amenable to the protein transfer mediated incorporation of GPI-anchored antigens and cytokines [12,40,60]. Protein transfer allows us to incorporate multiple GPI-anchored antigens and adjuvants into lipid enveloped VLPs. The novelty of our vaccine is that the GPI-anchored antigen is fused with cytokine adjuvant and anchored to VLPs, which deliver antigen and cytokine adjuvant to antigen-presenting cells simultaneously for a better immune response. 

We demonstrated that a hybrid vaccine based on influenza VLPs induces effective immunity against SARS-CoV-2 and influenza viruses. Our vaccine platform is based on cytokine adjuvants linked to the VLPs that carry the antigens [12]. This approach delivers both antigens and biological adjuvants to the immune system simultaneously in a particulate form. We generated CHO-S cells expressing GPI-anchored RBD-GM-CSF fusion protein and GPI-IL-12, purified the proteins and incorporated them onto influenza VLPs to develop the hybrid vaccine. Administration of the hybrid vaccine via either subcutaneous or intramuscular routes induced comparable levels of antibody response. Since soluble GPI-RBD-GM-CSF induced antibody response, but not the recombinant RBD-His tag, our results suggest that GM-CSF in the fusion protein acts as an adjuvant. Our approach to using cytokine adjuvants targets the antigen to APCs via their receptors and also allows the cytokines to enhance APC maturation. GM-CSF alone acting as an adjuvant in our GPI-RBD-GM-CSF fusion protein can induce a robust antibody response. Interestingly, the antibody response induced by the purified fusion protein is primarily the non-neutralizing IgG1 isotype, whereas fusion protein delivered using VLPs induced a neutralizing IgG2a and IgG1 mixed isotype antibody response. Our results are consistent with the recent report by the Bjorkman laboratory [44] and our previous studies on tumor antigens [40], demonstrating the use of VLP as a delivery vehicle for antigens to induce a protective immune response. The hybrid vaccine also induced neutralizing antibodies against influenza A/PR8 (H1N1), suggesting that this approach of delivering RBD on an influenza VLP along with cytokine adjuvants confers dual protection against both influenza A H1N1 and SARS-CoV-2 viruses.

Mice challenged with H1N1 live virus 3 months after the booster dose were still well protected, suggesting that anti-influenza antibody and T cell responses induced by hybrid vaccine are long-lasting. Neutralizing antibody titers against inactivated influenza A/PR8 (H1N1) are high even after 6 months of vaccination, also confirming the durability of the anti-influenza immune response induced by the hybrid vaccine. Mouse-adapted SARS-CoV-2 virus infection causes body weight loss but does not cause lethality in BALB/c mice [51]. This finding was observed within 3 days of infection in control mice that were vaccinated with VLP, and the hybrid vaccine prevented mice from losing weight. Lung virus titers were significantly decreased in mice that were vaccinated with a hybrid vaccine compared to plain VLP. This suggests that the hybrid vaccine containing GPI-RBD-GM-CSF with cytokine adjuvants confers protection from severe disease caused by SARS-CoV-2 infection. Administration of purified GPI-RBD-GM-CSF fusion protein without VLP also induced antibody response. However, the antibodies were not able to neutralize the live virus even though they were able to block ACE2 binding to RBD. The antibodies are mostly IgG1 in mice vaccinated with purified GPI-RBD-GM-CSF fusion protein alone, whereas a hybrid vaccine (VLP with GPI-RBD-GM-CSF fusion protein and GPI-IL-12) induced both IgG1 and IgG2a isotypes, and blocked SARS-CoV-2 virus infection. This suggests that the Th1 type response induced by the hybrid vaccine is more protective than the Th2 type response induced by the purified GPI-RBD-GM-CSF.

The protein transfer approach used here to prepare a hybrid vaccine allows the anchoring of these cytokines to the surface of the VLPs, which limits the systemic toxicity of the cytokines by acting as a depot at the site of vaccination. The physical linkage of adjuvant and antigen sources results in the presentation of the adjuvant and antigen simultaneously to the immune cells, leading to enhanced immune reactivity and increased vaccine efficacy. Such a physical linkage of antigen and adjuvants is more effective than an unconjugated antigen and adjuvant mixture [1,41]. In addition, IL-12 and GM-CSF target antigen-presenting cells, such as dendritic cells, by binding to IL-12 and GM-CSF receptors and enhancing antigen uptake and presentation, thereby enhancing subsequent T cell responses.

The limitation of the current study is that a comparative analysis of GPI-RBD-GM-CSF and GPI-RBD was not carried out to demonstrate the contribution of GM-CSF as an adjuvant in the VLP vaccine. Attempts to develop GPI-RBD in CHO S cells were not successful. However, the comparison of antibody response induced by purified GPI-RBD-GM-CSF molecule with RBD-His-Tag suggests that GM-CSF stimulated antibody production against RBD. Our studies suggest that it is possible to develop a two-in-one hybrid vaccine for influenza and SARS-CoV-2 viruses. In the future, when new variants of concern for SARS-CoV-2 arise, our hybrid vaccine can be modified to incorporate the RBD of new variants.

## 5. Conclusions

In summary, our results demonstrate that influenza VLP-based delivery of SARS-CoV-2 RBD protein in combination with cytokine adjuvants can be used as a platform to develop multivalent vaccines targeting the variant strains of viruses which are currently observed in the ongoing SARS-CoV-2 pandemic. Our fusion protein vaccine design also allows for the creation of fusion proteins with new variant sequences and quickly purify them using anti-GM-CSF mAb affinity chromatography. Further, the use of immobilized cytokines as adjuvants will provide a safer way to induce anti-viral immunity with minimal side effects.

## 6. Patents

Title: Composition and methods for detecting and treating a SARS-CoV-2 infection (patent filed).

## Data Availability

All data are available in the main text or supplementary materials.

## Supplementary Materials

The following supporting information can be downloaded at: https://www.mdpi.com/article/10.3390/vaccines10060944/s1, Figure S1. Purified GPI-RBD-GM-CSF fusion protein blocks binding of MM57 mAb to CHO-S cells expressing GPI-RBD-GM-CSF. Figure S2. Purified GPI-RBD-GM-CSF fusion protein and VLP vaccine incorporated with GPI-RBD-GM-CSF induce BMDC proliferation. Figure S3. Design for VLP vaccination in BALB/c mice. Figure S4. Booster dose induces high titers of anti-RBD antibody. Figure S5. VLP vaccine induces comparable levels of antibody against RBD. Figure S6. Hybrid vaccine induces RBD specific antibody response. Figure S7. VLP vaccine-induced antibody blocks ACE-2 binding to RBD. Figure S8. Hybrid vaccine but not the purified RBD-GM-CSF induces SARS-CoV-2 neutralizing antibody. Figure S9. GPI-RBD-GM-CSF fusion protein but not RBD induces antibody response against RBD. Figure S10. Hybrid vaccine induces antibody response against influenza VLP antigens. Figure S11. Hybrid vaccine induces antibody response against RBD-GM-CSF and influenza VLP antigens.
