# Supplementary material for: Influenza Virus-like Particle-Based Hybrid Vaccine Containing RBD Induces Immunity against Influenza and SARS-CoV-2 Viruses
Source: Vaccines (Basel). 2022 Jun 14;10(6):944. doi: 10.3390/vaccines10060944 (PMC9230705; doi:10.3390/vaccines10060944)
Supplement: Supplementary file 1 [file vaccines-10-00944-s001.zip › vaccines-1736201-supplementary.pptx]

## Slide 1
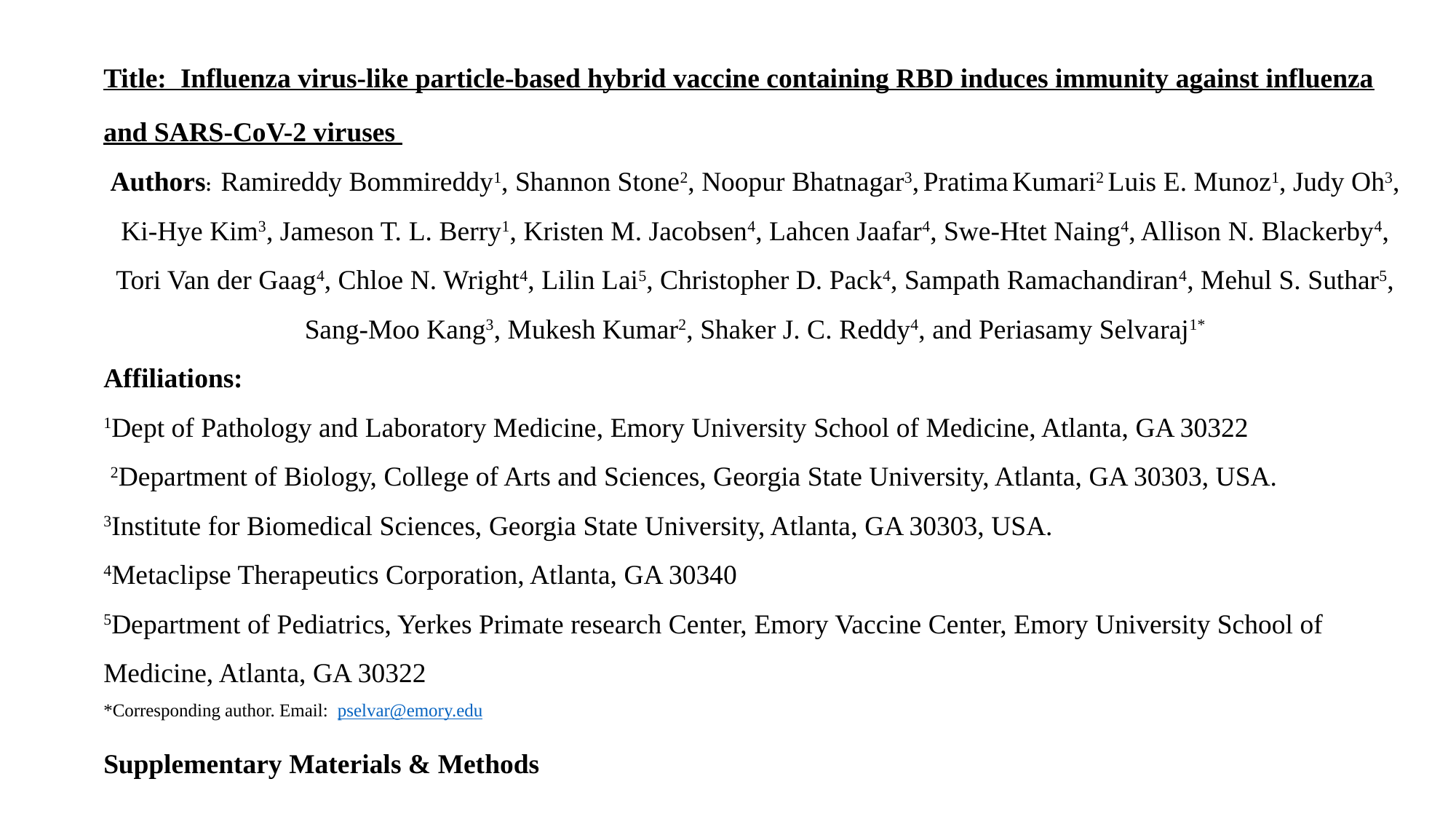

Title: Influenza virus-like particle-based hybrid vaccine containing RBD induces immunity against influenza and SARS-CoV-2 viruses
Authors: Ramireddy Bommireddy1, Shannon Stone2, Noopur Bhatnagar3, Pratima Kumari2 Luis E. Munoz1, Judy Oh3, Ki-Hye Kim3, Jameson T. L. Berry1, Kristen M. Jacobsen4, Lahcen Jaafar4, Swe-Htet Naing4, Allison N. Blackerby4, Tori Van der Gaag4, Chloe N. Wright4, Lilin Lai5, Christopher D. Pack4, Sampath Ramachandiran4, Mehul S. Suthar5, Sang-Moo Kang3, Mukesh Kumar2, Shaker J. C. Reddy4, and Periasamy Selvaraj1*
Affiliations:
1Dept of Pathology and Laboratory Medicine, Emory University School of Medicine, Atlanta, GA 30322
 2Department of Biology, College of Arts and Sciences, Georgia State University, Atlanta, GA 30303, USA.
3Institute for Biomedical Sciences, Georgia State University, Atlanta, GA 30303, USA.
4Metaclipse Therapeutics Corporation, Atlanta, GA 30340
5Department of Pediatrics, Yerkes Primate research Center, Emory Vaccine Center, Emory University School of Medicine, Atlanta, GA 30322
*Corresponding author. Email: pselvar@emory.edu
Supplementary Materials & Methods

## Slide 2
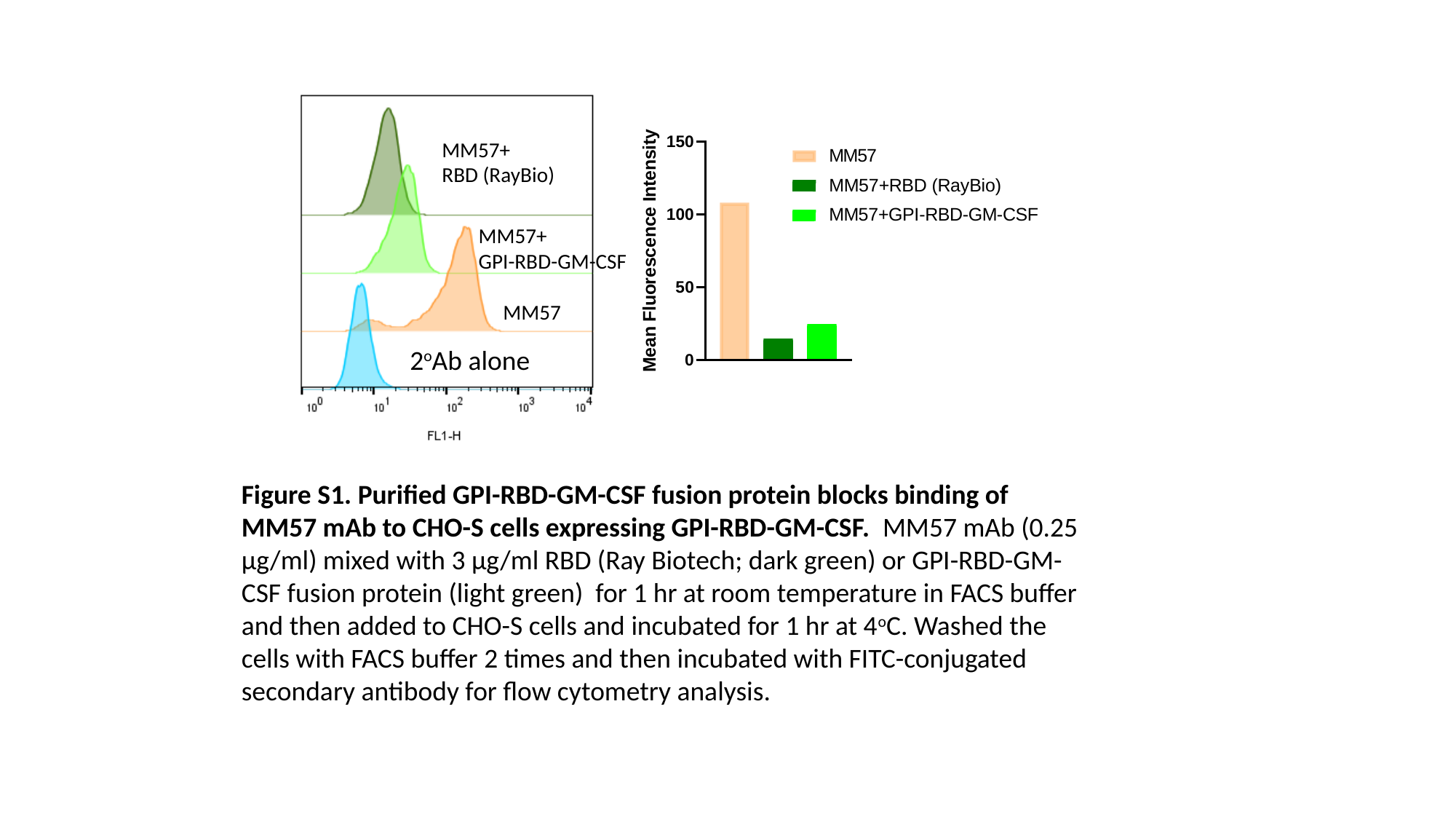

MM57+
RBD (RayBio)
MM57+
GPI-RBD-GM-CSF
MM57
2oAb alone
Figure S1. Purified GPI-RBD-GM-CSF fusion protein blocks binding of MM57 mAb to CHO-S cells expressing GPI-RBD-GM-CSF. MM57 mAb (0.25 µg/ml) mixed with 3 µg/ml RBD (Ray Biotech; dark green) or GPI-RBD-GM-CSF fusion protein (light green) for 1 hr at room temperature in FACS buffer and then added to CHO-S cells and incubated for 1 hr at 4oC. Washed the cells with FACS buffer 2 times and then incubated with FITC-conjugated secondary antibody for flow cytometry analysis.

## Slide 3
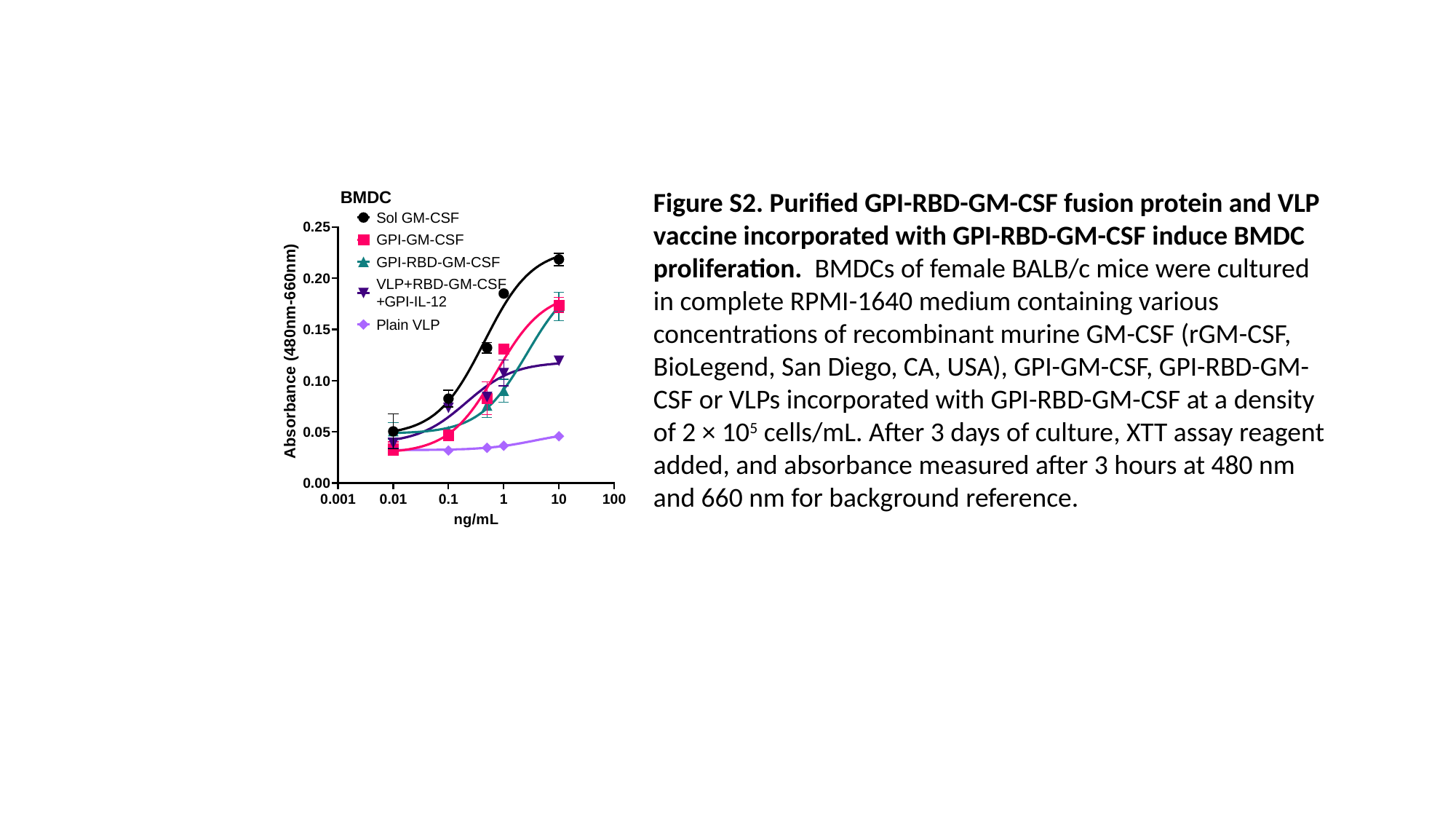

Figure S2. Purified GPI-RBD-GM-CSF fusion protein and VLP vaccine incorporated with GPI-RBD-GM-CSF induce BMDC proliferation. BMDCs of female BALB/c mice were cultured in complete RPMI-1640 medium containing various concentrations of recombinant murine GM-CSF (rGM-CSF, BioLegend, San Diego, CA, USA), GPI-GM-CSF, GPI-RBD-GM-CSF or VLPs incorporated with GPI-RBD-GM-CSF at a density of 2 × 10^5^ cells/mL. After 3 days of culture, XTT assay reagent added, and absorbance measured after 3 hours at 480 nm and 660 nm for background reference.

## Slide 4
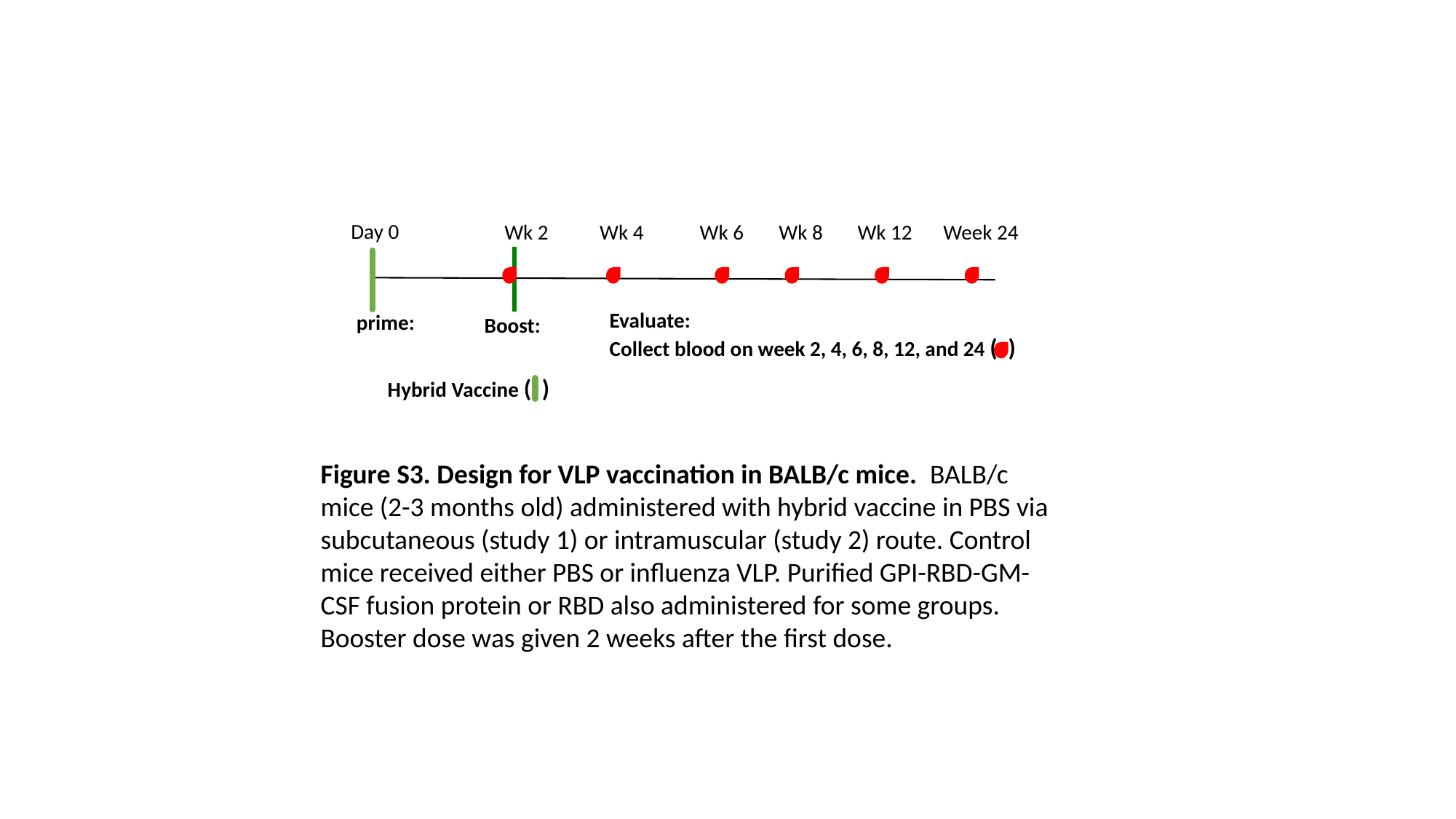

Day 0
prime:
Boost:
Evaluate:
Collect blood on week 2, 4, 6, 8, 12, and 24 ( )
Hybrid Vaccine ( )
Wk 2
Wk 4
Wk 6
Wk 8
Wk 12
Week 24
Figure S3. Design for VLP vaccination in BALB/c mice. BALB/c mice (2-3 months old) administered with hybrid vaccine in PBS via subcutaneous (study 1) or intramuscular (study 2) route. Control mice received either PBS or influenza VLP. Purified GPI-RBD-GM-CSF fusion protein or RBD also administered for some groups. Booster dose was given 2 weeks after the first dose.

## Slide 5
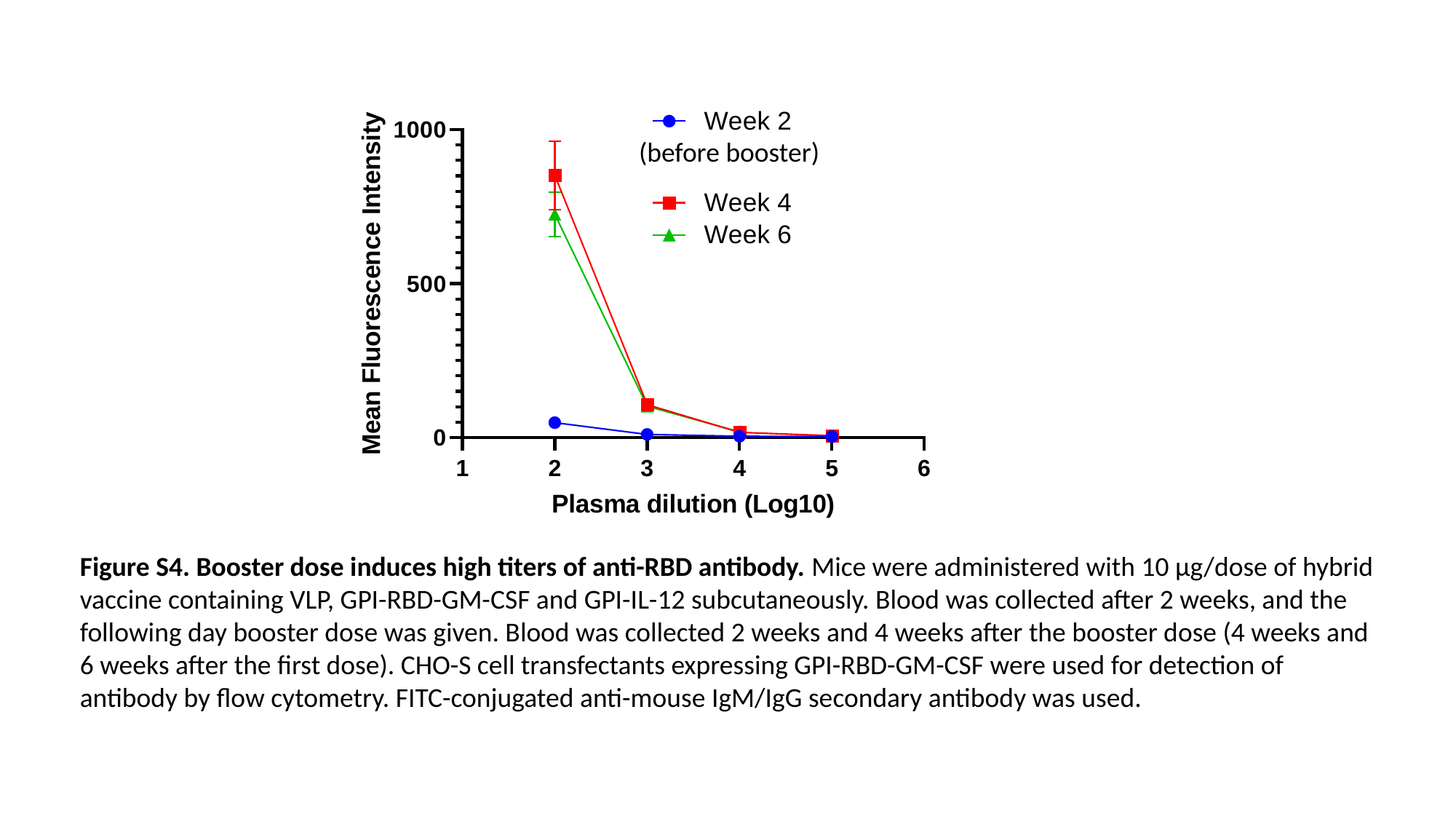

(before booster)
Figure S4. Booster dose induces high titers of anti-RBD antibody. Mice were administered with 10 µg/dose of hybrid vaccine containing VLP, GPI-RBD-GM-CSF and GPI-IL-12 subcutaneously. Blood was collected after 2 weeks, and the following day booster dose was given. Blood was collected 2 weeks and 4 weeks after the booster dose (4 weeks and 6 weeks after the first dose). CHO-S cell transfectants expressing GPI-RBD-GM-CSF were used for detection of antibody by flow cytometry. FITC-conjugated anti-mouse IgM/IgG secondary antibody was used.

## Slide 6
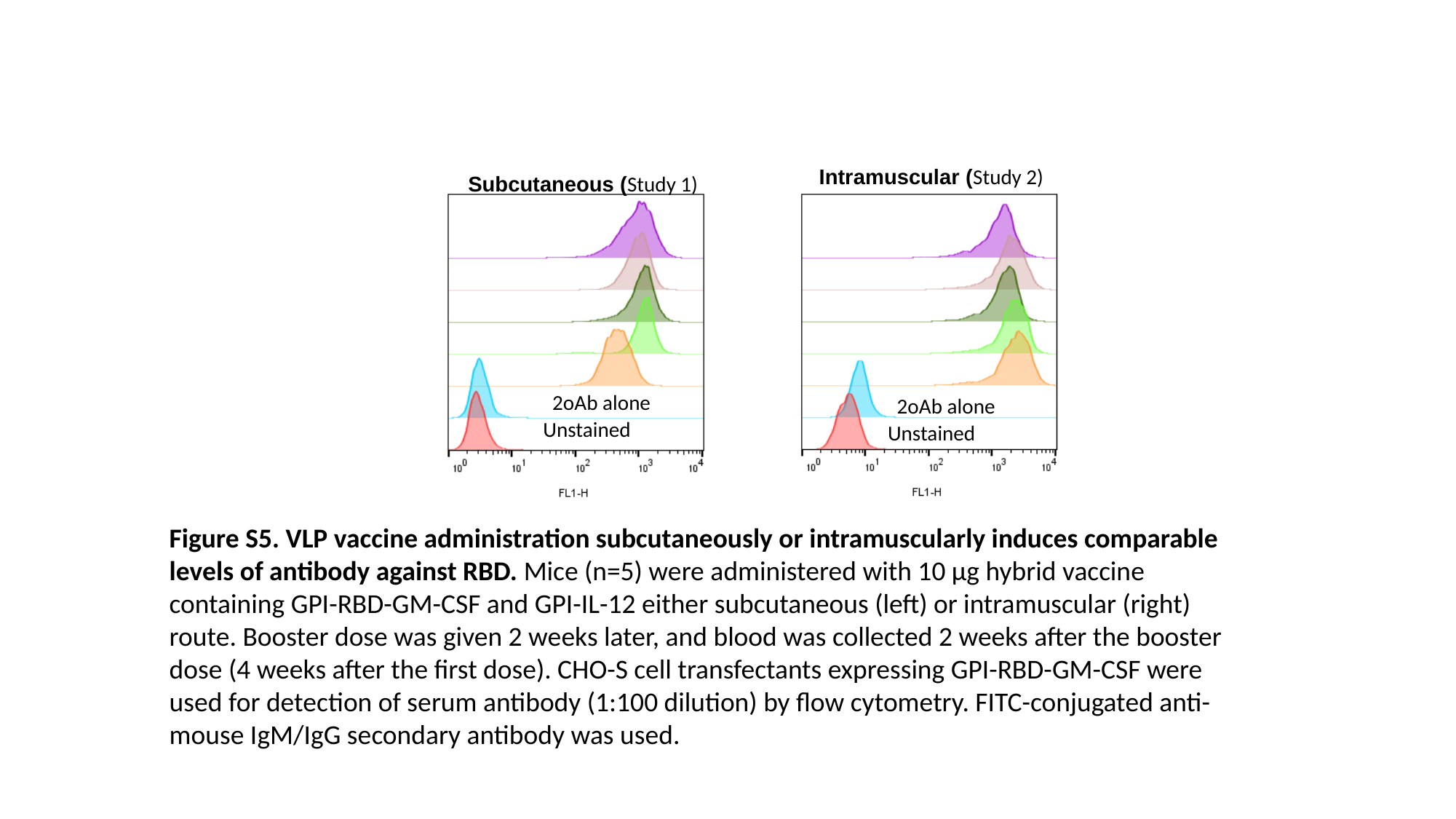

Intramuscular (Study 2)
Subcutaneous (Study 1)
2oAb alone
2oAb alone
Unstained
Unstained
Figure S5. VLP vaccine administration subcutaneously or intramuscularly induces comparable levels of antibody against RBD. Mice (n=5) were administered with 10 µg hybrid vaccine containing GPI-RBD-GM-CSF and GPI-IL-12 either subcutaneous (left) or intramuscular (right) route. Booster dose was given 2 weeks later, and blood was collected 2 weeks after the booster dose (4 weeks after the first dose). CHO-S cell transfectants expressing GPI-RBD-GM-CSF were used for detection of serum antibody (1:100 dilution) by flow cytometry. FITC-conjugated anti-mouse IgM/IgG secondary antibody was used.

## Slide 7
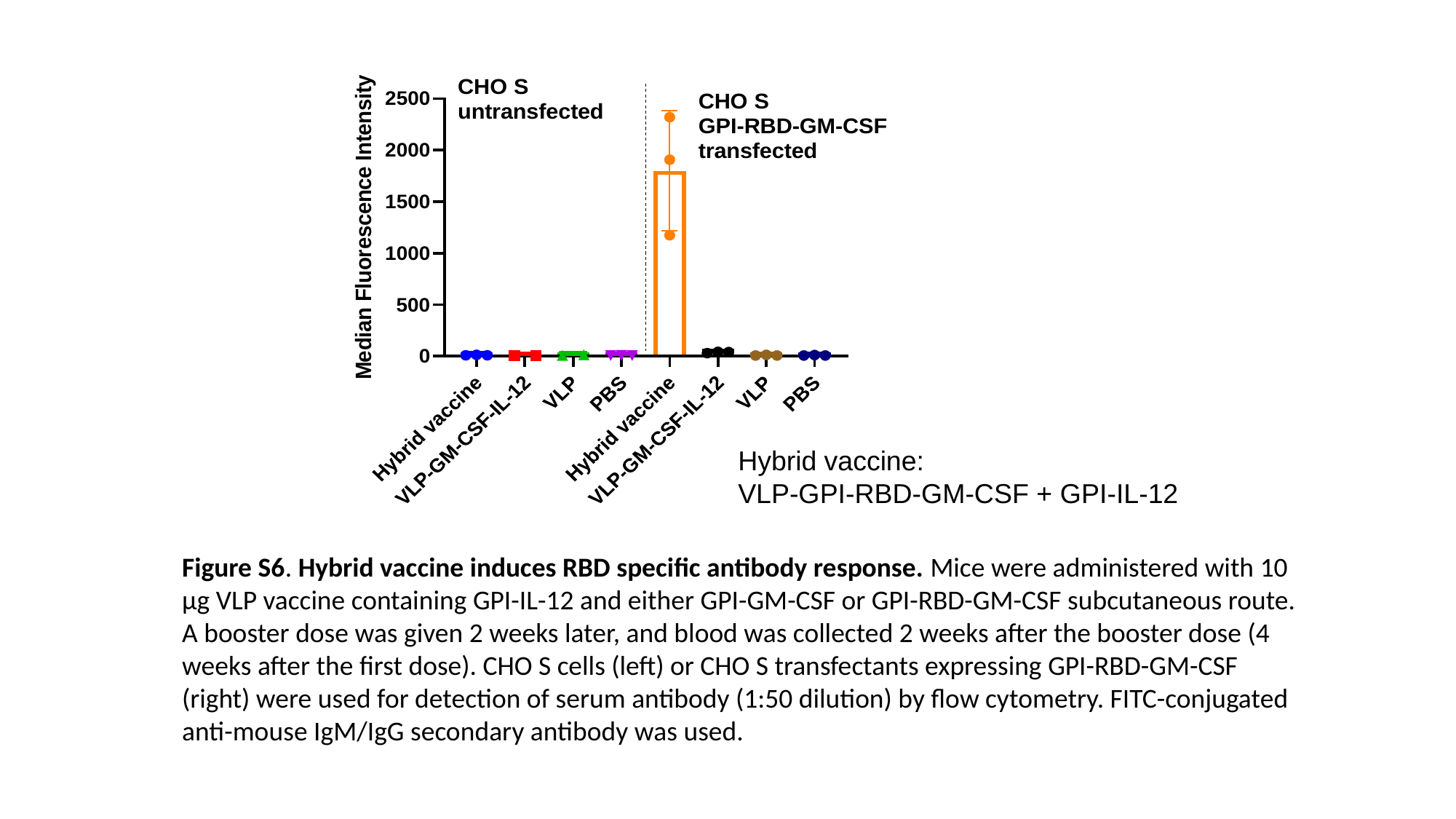

Hybrid vaccine:
VLP-GPI-RBD-GM-CSF + GPI-IL-12
Figure S6. Hybrid vaccine induces RBD specific antibody response. Mice were administered with 10 µg VLP vaccine containing GPI-IL-12 and either GPI-GM-CSF or GPI-RBD-GM-CSF subcutaneous route. A booster dose was given 2 weeks later, and blood was collected 2 weeks after the booster dose (4 weeks after the first dose). CHO S cells (left) or CHO S transfectants expressing GPI-RBD-GM-CSF (right) were used for detection of serum antibody (1:50 dilution) by flow cytometry. FITC-conjugated anti-mouse IgM/IgG secondary antibody was used.

## Slide 8
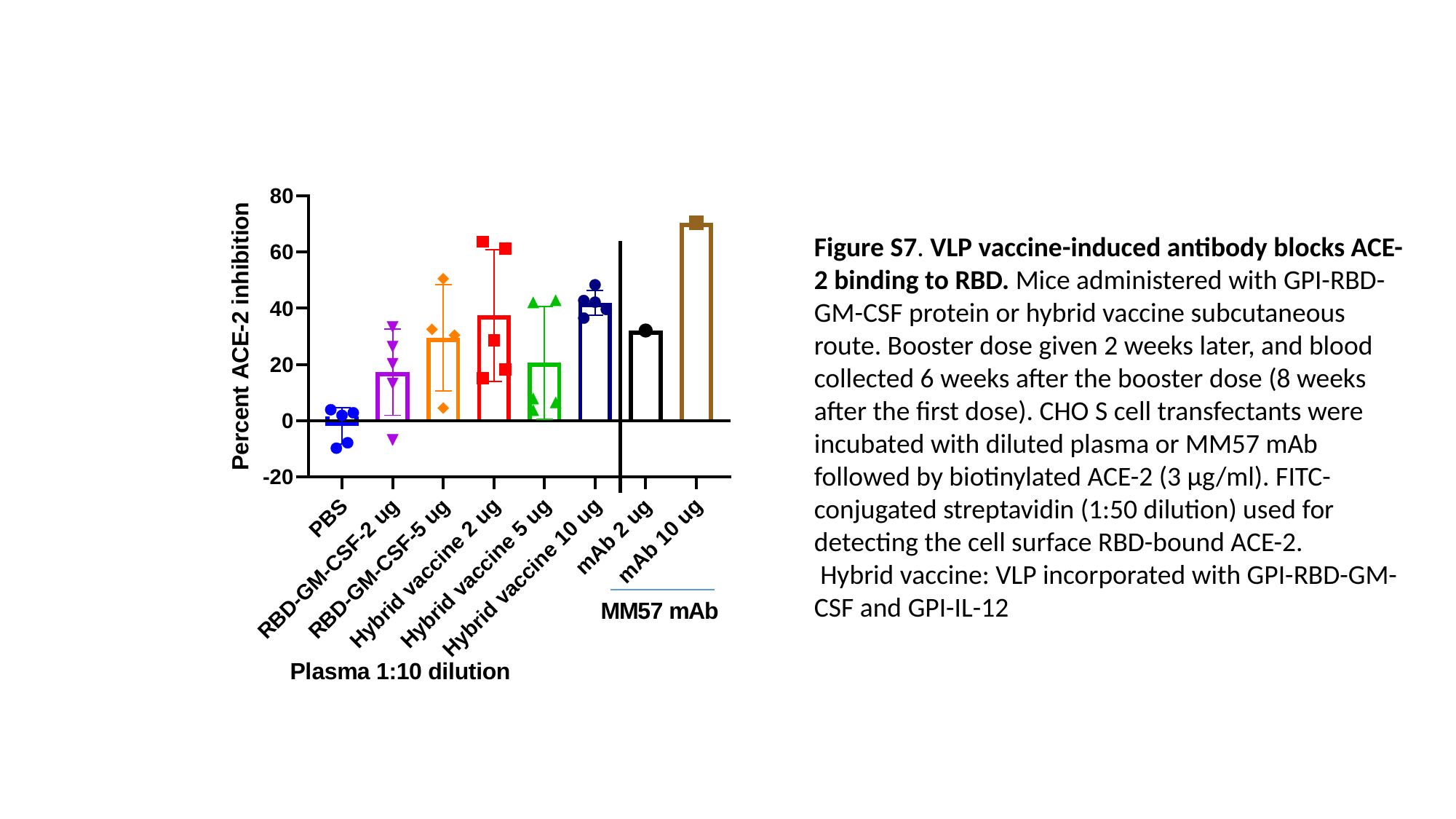

Figure S7. VLP vaccine-induced antibody blocks ACE-2 binding to RBD. Mice administered with GPI-RBD-GM-CSF protein or hybrid vaccine subcutaneous route. Booster dose given 2 weeks later, and blood collected 6 weeks after the booster dose (8 weeks after the first dose). CHO S cell transfectants were incubated with diluted plasma or MM57 mAb followed by biotinylated ACE-2 (3 µg/ml). FITC-conjugated streptavidin (1:50 dilution) used for detecting the cell surface RBD-bound ACE-2.
 Hybrid vaccine: VLP incorporated with GPI-RBD-GM-CSF and GPI-IL-12

## Slide 9
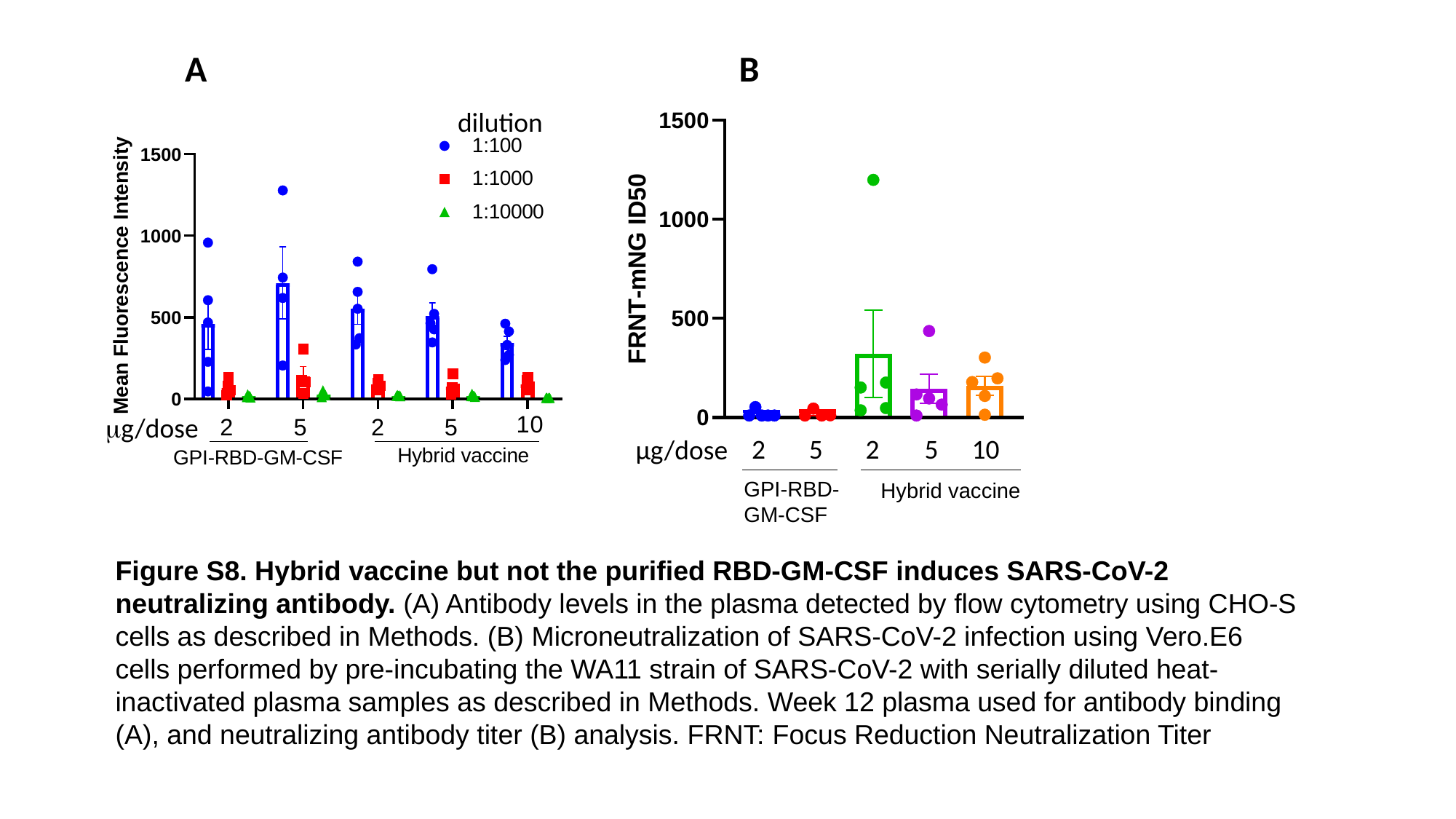

A
B
dilution
mg/dose
2
5
2
5
10
µg/dose
GPI-RBD-GM-CSF
Hybrid vaccine
Figure S8. Hybrid vaccine but not the purified RBD-GM-CSF induces SARS-CoV-2 neutralizing antibody. (A) Antibody levels in the plasma detected by flow cytometry using CHO-S cells as described in Methods. (B) Microneutralization of SARS-CoV-2 infection using Vero.E6 cells performed by pre-incubating the WA11 strain of SARS-CoV-2 with serially diluted heat-inactivated plasma samples as described in Methods. Week 12 plasma used for antibody binding (A), and neutralizing antibody titer (B) analysis. FRNT: Focus Reduction Neutralization Titer

## Slide 10
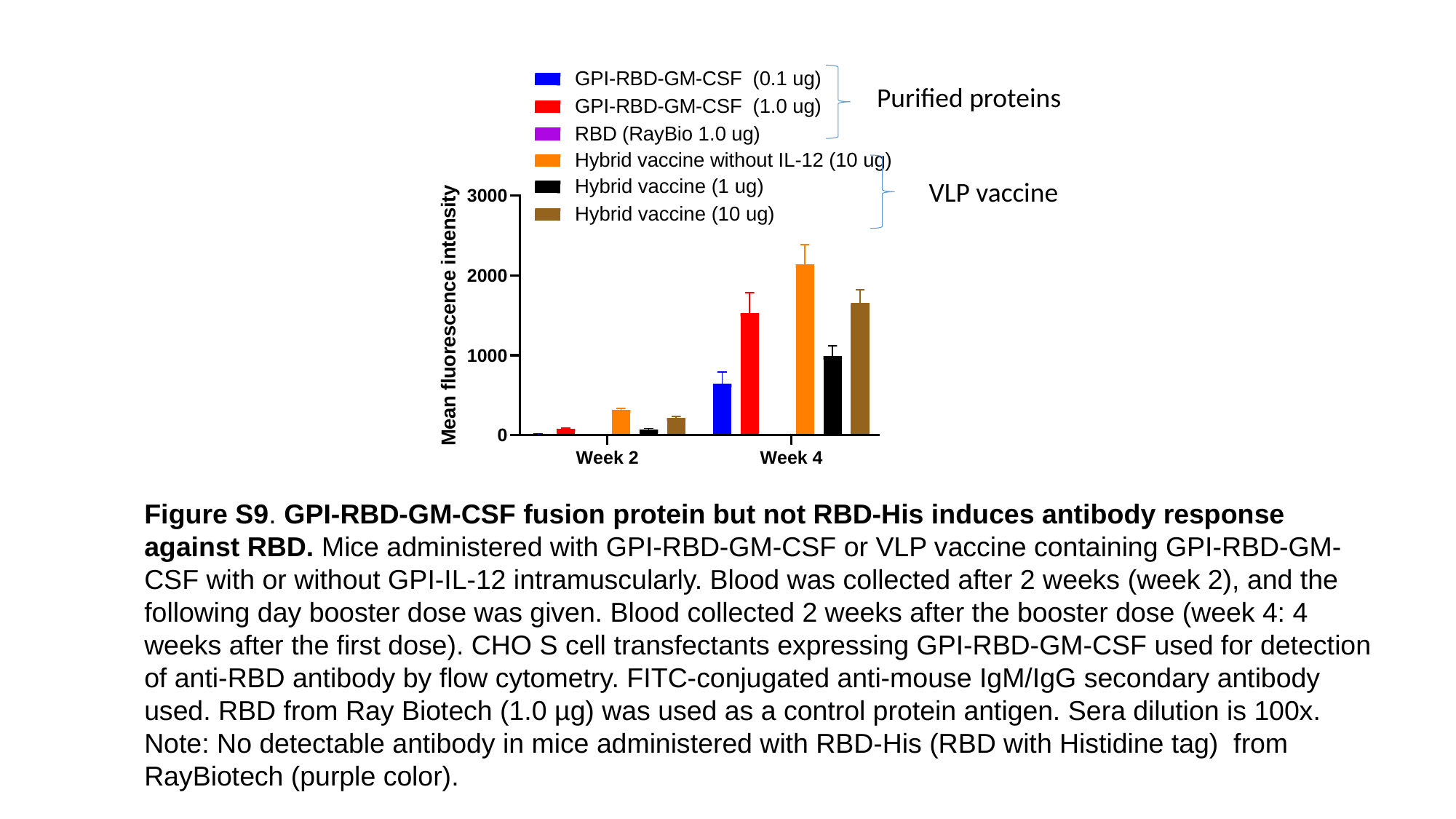

Purified proteins
VLP vaccine
Figure S9. GPI-RBD-GM-CSF fusion protein but not RBD-His induces antibody response against RBD. Mice administered with GPI-RBD-GM-CSF or VLP vaccine containing GPI-RBD-GM-CSF with or without GPI-IL-12 intramuscularly. Blood was collected after 2 weeks (week 2), and the following day booster dose was given. Blood collected 2 weeks after the booster dose (week 4: 4 weeks after the first dose). CHO S cell transfectants expressing GPI-RBD-GM-CSF used for detection of anti-RBD antibody by flow cytometry. FITC-conjugated anti-mouse IgM/IgG secondary antibody used. RBD from Ray Biotech (1.0 µg) was used as a control protein antigen. Sera dilution is 100x.
Note: No detectable antibody in mice administered with RBD-His (RBD with Histidine tag) from RayBiotech (purple color).

## Slide 11
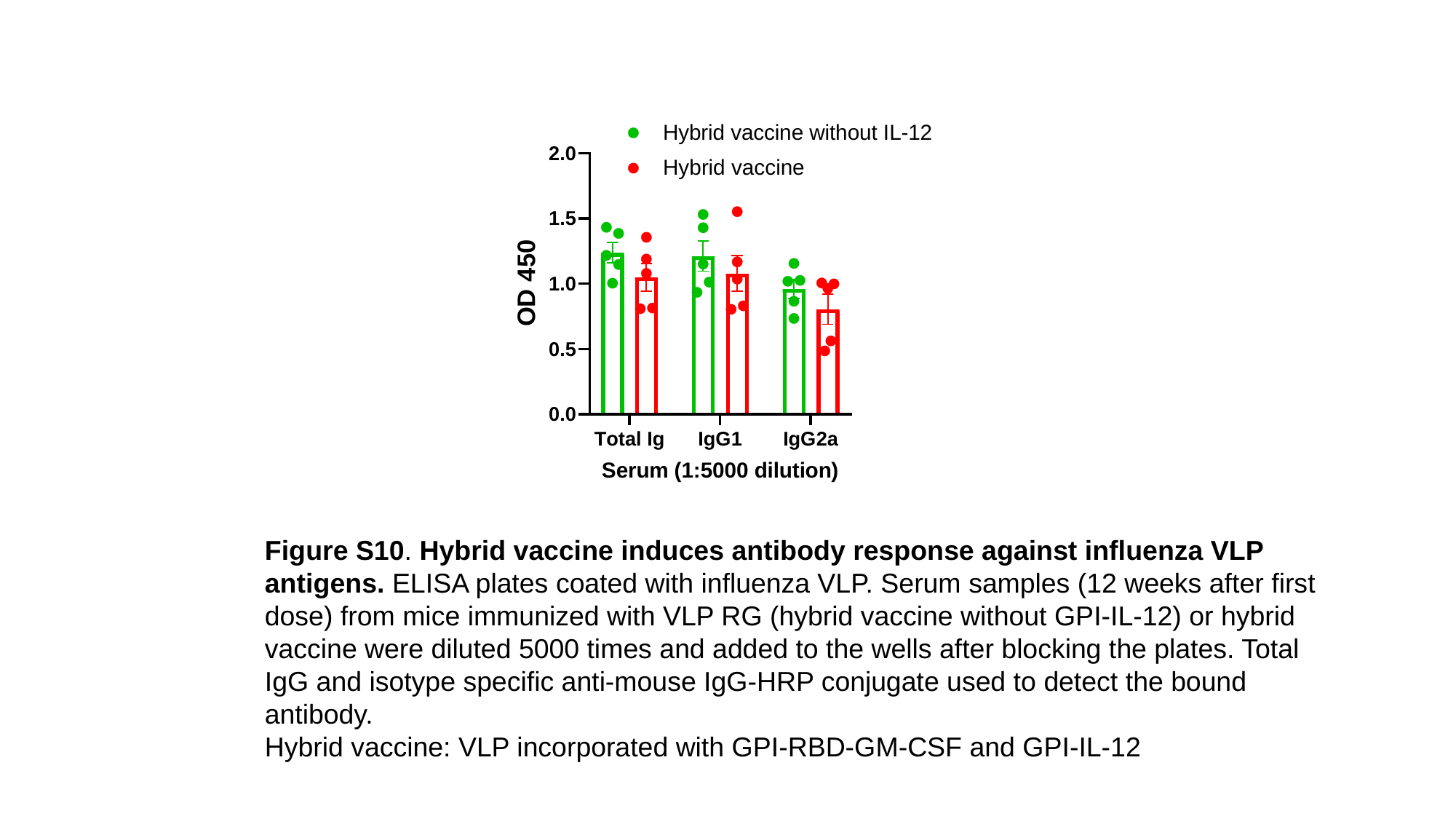

Figure S10. Hybrid vaccine induces antibody response against influenza VLP antigens. ELISA plates coated with influenza VLP. Serum samples (12 weeks after first dose) from mice immunized with VLP RG (hybrid vaccine without GPI-IL-12) or hybrid vaccine were diluted 5000 times and added to the wells after blocking the plates. Total IgG and isotype specific anti-mouse IgG-HRP conjugate used to detect the bound antibody.
Hybrid vaccine: VLP incorporated with GPI-RBD-GM-CSF and GPI-IL-12

## Slide 12
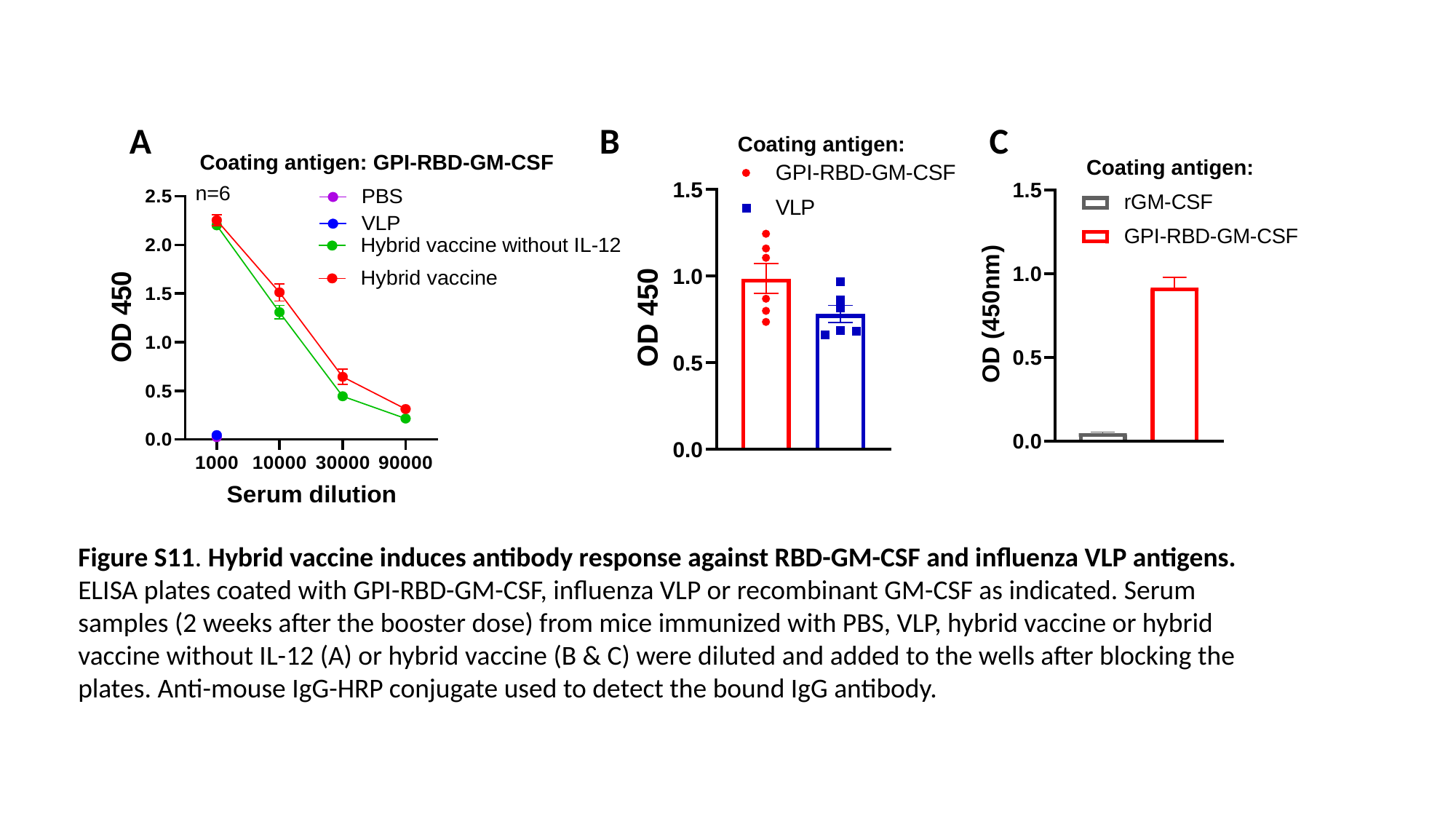

A
B
C
Coating antigen:
Coating antigen: GPI-RBD-GM-CSF
Coating antigen:
Figure S11. Hybrid vaccine induces antibody response against RBD-GM-CSF and influenza VLP antigens. ELISA plates coated with GPI-RBD-GM-CSF, influenza VLP or recombinant GM-CSF as indicated. Serum samples (2 weeks after the booster dose) from mice immunized with PBS, VLP, hybrid vaccine or hybrid vaccine without IL-12 (A) or hybrid vaccine (B & C) were diluted and added to the wells after blocking the plates. Anti-mouse IgG-HRP conjugate used to detect the bound IgG antibody.
